# Effects of progestogen neurosteroids on locomotor activity in zebrafish embryos and larvae

**DOI:** 10.1007/s10695-025-01519-6

**Published:** 2025-05-29

**Authors:** Mandarin Mathouchanh, Charles A. Lessman

**Affiliations:** Department of Biological Sciences, The University of Memphis, Memphis, TN 38152 USA

**Keywords:** Progesterone, Deoxycorticosterone, Behavior, Neuromuscular system, GABA_A_ receptor, Membrane progesterone receptor

## Abstract

The steroid hormone progesterone (P4) and related compounds called progestogens are well known for their effects on the reproductive system. However, their physiological roles outside reproduction are less understood. Additionally, there is limited information on the toxicological repercussions of environmental exposure to exogenous progestogens and how such exposure might impact the development and survival of animals living in contaminated habitats. Two progesterone-based neurosteroids, allopregnanolone and tetrahydrodeoxycorticosterone (THDOC), are known to enhance γ-aminobutyric acid type A (GABA_A_) receptor activity, inducing neuronal hyperpolarization. In this study, locomotor parameters in zebrafish embryos and larvae were used as endpoints to assess the inhibitory effects of pregnane neurosteroids. Specifically, spontaneous tail coiling in embryos at 24 h post-fertilization (hpf) and swimming activity in larvae aged 120–168 hpf were evaluated. Motility assays in embryos showed that P4 increased spontaneous tail coiling, whereas larvae exhibited an anesthetic-like loss of motility. This effect was both age- and dose-dependent for P4, deoxycorticosterone (DOC), 5α-dihydroprogesterone, and the membrane progesterone receptor agonist, ORG-OD-02–0, but not for other steroids tested. Removal of the steroids after the onset of anesthesia led to recovery of motility in larvae, suggesting that the observed effects are not due to a non-specific toxicity. Our results suggest that P4 targets the mPR, which acts in association with the GABA_A_ receptor to mediate the loss of locomotor behavior. This study provides further insight into how neuroactive compounds can affect locomotor behaviors during early developmental stages in nonmammalian species.

**Supplementary Information:**

The online version contains supplementary material available at 10.1007/s10695-025-01519-6.

## Introduction

Sex steroids are hormones primarily known for their roles in the reproductive system. However, a myriad of data have emerged that show steroid hormones also play important roles beyond reproduction. For example, progesterone (P4) and its metabolites (collectively called progestogens), which are synthesized in neurons and radial glial cells in the brain, are known as pregnane neurosteroids (Reddy 2010). These neuroactive steroids act as powerful modulators of neuronal excitability and induce anesthetic-like properties in vertebrates via rapid nongenomic mechanisms. They also have significant roles in brain function and neuroprotection (Selye 1941; Komisaruk et al. 1967; Majewska et al., 1986; Kokate et al. 1994; Ghoumari et al. 2005; Charalampopoulos et al. 2008; Singh and Su 2013; Diotel et al. 2018). Therefore, in addition to the genomic effects mediated by intracellular steroid receptors, certain steroids can rapidly alter neuronal activity by binding to membrane-bound receptors (Table 1). Neurosteroids primarily regulate brain function by acting as positive or negative modulators of several neurotransmitter receptors, including γ-aminobutyric acid type A (GABA_A_), *N*-methyl-d-aspartate (NMDA), α-amino-3-hydroxy-5-methyl-4-isoxazolepropionic acid (AMPA), and acetylcholine (ACh) receptors (Wojtal et al. 2006; Mitchell et al. 2008; Reddy 2010; Borowicz et al. 2011; Parakala et al. 2019).

**Table 1:** Examples of receptors targeted by progestogens

Receptor	Location	Agonists/PAM/NAM	Antagonists
nPR	Nucleus	P4, progestogens	RU486
mPR	Cell membrane	P4, ORG-OD-02–0	None reported
GR	Nucleus	Corticoids	RU486
GABAA	Neuronal membrane	GABA, neurosteroids	PTX
PGRMC1	Cell membrane, endoplasmic reticulum	P4	AG205

*PAM*, positive allosteric modulator; *NAM*, negative allosteric modulator; *PTX*, picrotoxin

Progestogens such as allopregnanolone and tetrahydrodeoxycorticosterone (THDOC) are neurosteroids that act as powerful positive allosteric modulators (PAMs) of the GABA_A_ receptor, enhancing Cl^−^ influx (Majewska et al. 1986; Harrison and Simmonds 1984; Barker et al. 1987; Kokate et al. 1994; Belelli and Lambert 2005; Agís-Balboa et al. 2006; Herd et al. 2007). In zebrafish, the GABA_A_ receptor is a heteropentameric ligand-gated ion channel composed of 2 α subunits, 2 β subunits, and a fifth subunit belonging to one of several classes: γ, δ, π, ζ, or ρ. Zebrafish have been found to possess 23 GABA_A_ receptor subunits: α1, α2a, α2b, α3–5, α6a, α6b, β1–4, γ1–3, δ, π, ζ, ρ1, ρ2a, ρ2b, ρ3a, and ρ3b (Monesson-Olson et al. 2018; Sadamitsu et al. 2021). Extrasynaptic GABA_A_ receptors that facilitate tonic inhibition typically contain δ subunits (Farrar et al. 1999; Bryson et al. 2020; Sadamitsu et al. 2021). Activation of these receptors leads to prolonged inhibition by responding to ambient GABA that has escaped the synaptic cleft (Farrant and Nusser 2005; Lee and Maguire 2014). This contrasts with phasic inhibition, which is faster and mediated by synaptic GABA_A_ receptors containing the γ subunit (Farrant and Nusser 2005). Neurosteroids are believed to have a higher affinity for δ-containing GABA_A_ receptors, exerting their effects primarily through tonic inhibition to reduce neuronal excitability (Mihalek et al. 1999; Wohlfarth et al. 2002; Stell et al. 2003). In zebrafish, cells expressing GABA_A_ receptors are distributed throughout the brain by 16 h post-fertilization (hpf) (Doldán et al. 1999; Song et al. 2017).

Phosphorylation of the GABA_A_ receptor has been reported to be facilitated by the membrane progesterone receptor (mPR) (Parakala et al. 2019). mPRs are localized on the cell surface and mediate rapid progestogen activity through nongenomic intracellular signaling pathways, similar to G-protein coupled receptors (GPCRs) (Ramirez and Zheng 1996; Thomas and Pang 2012; Ryu et al. 2017; Thomas 2022; Thomas et al. 2022). Additionally, mPRs are highly expressed in the brain, where they serve as targets for pregnane neurosteroids and couple to either *G*_i/o_ or *G*_s_ signaling pathways (Zhu et al. 2003a, b; Tang et al. 2005; Kelder et al. 2010; Thomas and Pang 2012; Meffre et al. 2013). Despite being 7-pass transmembrane proteins that utilize G-proteins, mPRs do not belong to the GPCR superfamily; instead, they are members of the progestin and adipoQ receptor (PAQR) family (Tang et al. 2005; Thomas 2012; Thomas and Pang 2012). Activation of zebrafish mPRα and mPRβ inhibits adenylyl cyclase activity and significantly decreases cyclic adenosine monophosphate (cAMP) production (Hanna et al. 2006). Although mPR activation has been shown to induce GABA_A_ receptor phosphorylation in mammalian cells (Parakala et al. 2019), it remains unknown whether this metabotropic effect leads to behavioral inhibition of locomotor activity in zebrafish.

The zebrafish is a valuable model for studying the effects of different compounds on vertebrate neural pathways. Adults can spawn large numbers of fertilized, transparent embryos that develop rapidly and synchronously, making them highly suitable for medium- to high-throughput chemical screening (Kimmel et al. 1995; Lessman 2011). Toxicological endpoints assessed in these screenings include embryonic malformations, lethality, and behavioral assessments, which are often used as indicators of neurotoxicity (Selderslaghs et al. 2010; Tierney 2011; Hill et al. 2021). Zebrafish are also valuable due to well-characterized patterns of locomotion across developmental stages (Saint-Amant and Drapeau 1998). Studies have demonstrated that exposure to endocrine-disrupting chemicals, even at environmental levels, can inhibit swimming behavior in larval zebrafish (Fraser et al. 2017; Gu et al. 2020; Dong et al. 2023). Given that GABA_A_ receptors are phosphorylated following mPR activation in mammalian models (Parakala et al. 2019), it is important to characterize the neural and behavioral effects of progestogens in fish species.

This study aimed to investigate whether progestogen-induced inhibition of locomotor behaviors in embryonic and larval zebrafish is mediated by specific steroid hormone receptors, such as the glucocorticoid receptor (GR), nuclear progesterone receptor (nPR), and mPR, either independently or in association with GABA_A_ receptors. Here, we present our observations on the behavioral effects of topical progesterone using motility assays in embryonic and larval zebrafish.

## Materials and methods

### Animals and housing

Adult male and female wild-type (WT) AB strain zebrafish (*Danio rerio*) maintained from a culture established at the University of Memphis were used to spawn embryos for this study. All adult fish are raised on a 14:10-h light:dark photoperiod cycle, with lights on from 8 am to 10 pm. Temperature was maintained at 28 ± 1 °C. All fish received daily water changes with a combination of tap water and reverse osmosis (RO) water, along with sodium thiosulfate to remove chlorine and an ammonia remover solution (Amquel, Aquatic Eco Systems, Inc.). Fish were fed once daily with commercially available flaked fish food (TetraMin) and freeze-dried brine shrimp (Aquatic Eco Systems, Inc.). Fish care was in accordance with approved Institutional Animal Care and Use Committee protocols at the University of Memphis. For all experiments described, embryos were staged according to the methods described by Kimmel et al. (1995).

### Chemicals and materials

Stock solutions for P4, pregnenolone (P5) (Steraloids), cortisone, hydrocortisone, 17α-dihydroxyprogesterone (17α-HP), 17α,20β-dihydroxyprogesterone (17α,20β-DHP), deoxycorticosterone (DOC) (Sigma), ORG-OD-02–0 (ORG) (Axon Inc.), 5α-dihydroprogesterone (5α-DHP), picrotoxin (PTX), finasteride, RU486, and AG205 (Sigma) were made by dissolving with a steroid vehicle composed of a 1:1 ratio of propylene glycol and ethanol (all steroids) or dimethyl sulfoxide (DMSO) (AG205). Stock solutions were stored in the refrigerator at 4 °C until use. Epson Scanners, Zeiss dissecting microscope with attached camera (Amscope), standalone camera system (Elikliv), and the imaging software ImageJ (Fiji) were utilized to assess embryonic motility upon treatment with steroid hormones.

### Embryo collection

Healthy adult male and female WT zebrafish mating pairs were placed into 1 L spawning tanks (Aquaneering Inc.) filled with system water the day before egg collection. Ovulation and fertilization typically occur when the lights turn on in the morning (8 am), when fish are stimulated to mate. Embryos were collected soon after the onset of light by straining the spawning tank water through a mesh screen. These embryos were washed with egg water (dechlorinated water and reverse osmosis water containing one drop/L of 1% anti-fungal methylene blue solution) and transferred to a 100 × 15-m Petri dish filled with egg water. Embryos were checked for quality, and any unfertilized eggs or embryos with cleavage irregularities were removed via hand pipette. Debris found in the petri dish (adult waste products or leftover fish food) was discarded. Embryos were incubated in egg water at 28 ± 1 °C until exposure to test compounds. Multiple batches of embryos were collected from different mating pairs, and appropriate quality was decided by batches having 10% or fewer unfertilized eggs or dead eggs.

### Exposure to steroid hormones

Stocks of steroid hormones such as P4, P5, cortisone, hydrocortisone, 17α-HP, 17α,20β-DHP, DOC, ORG, and 5α-DHP were made at different concentrations through serial dilutions to use in each test treatment group. Other compounds, including PTX, finasteride, RU486, and AG205, were used in cotreatment groups to help delineate the receptors that may be responsible for motility effects. Healthy 5–7-dpf larvae (no sign of heart or yolk sac edemas, normal heart rate, and showing swimming behaviors) were randomly chosen and immersed individually into the wells of a 96-well plate filled with 200 µL of test solution per well, with 10 larvae per treatment. Although there is one zebrafish larva per well, there are 10 wells that contain the same concentration of steroid tested, totaling 10 biological replicates per treatment per experiment. For embryonic motility assays,10 embryos were immersed in each well of 8-well plates filled with 5 mL of test solution. The final concentration of the steroid vehicle (SV) in the control and test solutions was at most 1% (i.e., 0.5% ethanol and propylene glycol each).

To determine the lowest concentration of P4 that would elicit a locomotor response, zebrafish larvae were exposed to concentrations of P4 ranging from 0.1 to 10 µM. The high dose was used in subsequent experiments since acute exposures eliminated locomotor activity in larval-stage zebrafish without causing mortality. To confirm that the inhibitory effects on locomotor activity by P4 were not due to a non-specific toxic effect, images were captured as the larvae were immersed in 10 µM P4 for 2 h. The P4 solution was then removed, and larvae were washed with and immersed in untreated egg water. Image capture resumed to evaluate motility after the wash.

Several potential targets for the motility effects of progestogens were tested, including nPR, mPR, and GABA_A_. The possible role that nuclear steroid hormone receptors like the nPR and GR can play in reducing locomotor activity was determined using 10 and 30 µM P5, 30 µM 17α-HP (a ligand for the nPR), 1 µM RU486 (an antagonist to the nPR and partial antagonist to the GR), and 3 and 10 µM of the corticosteroids cortisone and cortisol (also called hydrocortisone). The role of the mPR in larval locomotor activity was investigated using the endogenous mPRα ligand 10 and 30 µM 17α,20β-DHP (which acts as a maturation-inducing steroid (MIS) of oocytes in the ovaries) and 3, 10, and 30 µM ORG (a specific mPR agonist). To assess how the GABA_A_ receptor can impact larval motility upon exposure to P4, larvae were treated with 3, 10, and 30 µM DOC (a mineralocorticoid and possible modulator for the GABA_A_ receptor), 0.5, 1, 2, 3, 4, 10, and 30 µM 5α-DHP (the immediate precursor to allopregnanolone, another PAM). Additionally, cotreatments using inhibitors were applied to reveal the role the GABA_A_ receptors play in mediating motility effects. These included 3, 10, and 30 µM finasteride (an inhibitor of the steroidogenic enzyme 5α-reductase), and 400 µM and 2 mM PTX (a GABA_A_ receptor channel blocker). Finasteride was used to evaluate the role that DOC plays in modulating the GABA_A_ receptor to induce anesthesia. The larvae were pre-treated with 10 µM finasteride for 30 min before the addition of P4, DOC, and 5α-DHP.

### Using time-lapse images to assess locomotor activity in zebrafish larvae

**Fig. 1:**
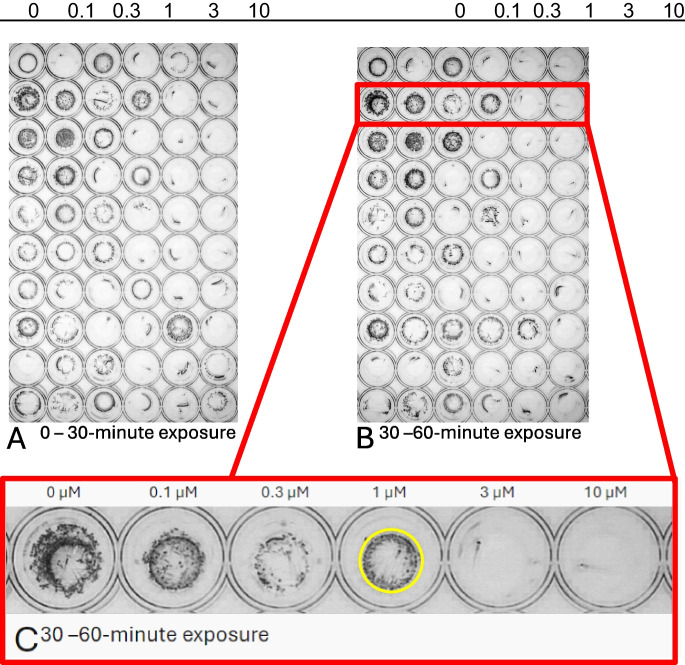
Z-projection of time-lapse stacks (5-s intervals) from a multi-well plate. Each well contains one zebrafish larva in 200 µL of treatment. Densitometry was performed in ImageJ: **A** 0–30-min interval; **B** 30–60-min interval. Progesterone (P4) concentrations are listed above each column. **C** Enlarged view of larvae from (**B**, row 2); the ROI is indicated by the yellow circle

Larval zebrafish motility was evaluated using the computer-aided screening (CAS) method as described previously (Lessman 2002, 2004; Lessman et al. 2010). Briefly, larvae were imaged at 2-min intervals using flatbed digital scanners set at 1200 dots per inch (dpi), 8-bit grayscale. For shorter time intervals, 96-well plates were placed on top of a light board and positioned under an Elikliv camera to record spontaneous swimming. Images were recorded every 5 s via the use of a Macro Scheduler program (mjtnet.com). A thermometer probe was kept near the plate to measure the temperature around the plate, which was maintained at 29 ± 1 °C by a space heater. Images collected were processed in ImageJ, in which stacks of images were made from different time intervals of image collection. These stacks were collapsed into a z-projection image to evaluate how locomotor activity was temporally affected during steroid exposure (Fig. 1). Locomotor activity was quantified by covering each well with a region of interest (ROI) in the z-projected images. The average pixel densities (APD) of the larvae within the ROI for each treatment group were measured and recorded. High APD values indicate high locomotor activity, as the larva will be in a different orientation within the well every time an image is captured and therefore fill in the spaces in the well. Low locomotor activity is represented by low APD values since most of the pixels captured within the ROI represent empty well space. This assay provided estimates of the sedative and anesthetic properties of the test compounds through quantification of locomotor activity.

### Using video recordings to measure spontaneous tail coiling behaviors in zebrafish embryos

Spontaneous tail coiling of embryonic zebrafish (24 hpf) upon acute P4 exposure was also analyzed. Healthy embryos were collected from stock, and 10 were placed in each well. At 22 hpf, embryos were exposed to 5 mL of a range of P4 concentrations. The plates were placed back into the incubator at 28 ± 1 °C for 2 h. By 24 hpf, the embryos were examined under a dissecting microscope at 12X magnification, and videos were recorded using an attached camera (AmScope) for 2 min per treatment at 2 frames per second. While recording the embryos using a high-speed camera at higher frames per second would allow for more thorough capturing of embryonic movement, we were limited by the camera available, storage size, and processing power of the computers. Although we found that 2 frames per second was sufficient to capture, if not the entire tail coiling behavior, then at least the beginning or end of one, indicating movement. Videos from each of these assays were imported into ImageJ, and movie files were analyzed frame by frame. To screen for motility effects, tail coiling was measured using average greyness values to quantify differences in pixel density within a set ROI. The ROI encompasses a quarter of the space taken up by the chorion that surrounds each embryo. As the embryo tumbles within the chorion due to muscle contractions resulting in spontaneous coiling behaviors associated with this stage of development, the orientation of the embryo within the chorion will change. This change is captured by the stationary ROI, allowing average greyness density within the ROI to be quantified. These values were recorded in Microsoft Excel, and substantial changes greater than 1 greyness unit indicated movement. When movement is detected, the videos are watched, and each coiling behavior is counted as 1 movement to reinforce the accuracy of the ROI data. The total number of spontaneous tail coiling behaviors was counted and recorded for each embryo in each treatment group.

### Statistical analysis

Steroid treatment and control groups contained ten biological replicates, with one embryo or larva in each replicate. For assessing the effect of P4 on spontaneous tail coiling behaviors in 24-hpf zebrafish embryos, videos were analyzed in ImageJ, and the coiling data were uploaded to GraphPad Prism 10. To evaluate the effects of progestogens on swimming behavior in 5–7 dpf zebrafish larvae, the APD of the fish was measured in each well for each treatment and recorded in Microsoft Excel. The APD data were then transferred to GraphPad Prism 10. A one-way analysis of variance (ANOVA) was performed to determine significance in motility between drug treatment groups and the steroid vehicle control. If an overall difference between groups was detected, post hoc analysis was performed using Tukey’s multiple-comparison test or Dunnett’s multiple-comparison test to compare the average number of spontaneous tail coiling of embryos or APD within specified treatments and co-treatment groups. *t* Test analysis was used to evaluate whether P4 had a non-specific toxic effect on zebrafish larvae. When comparing averages between groups, statistical significance was achieved if *p* < 0.05. All data are presented as the average ± standard deviation (SD), and statistically significant differences between the means are marked with asterisks: *p < 0.05, **p < 0.01, ***p < 0.001, and ****p < 0.0001.

## Results

### Exposure to progesterone reduces locomotor activity in 7-dpf zebrafish larvae in a time- and dose-dependent manner

**Fig. 2:**
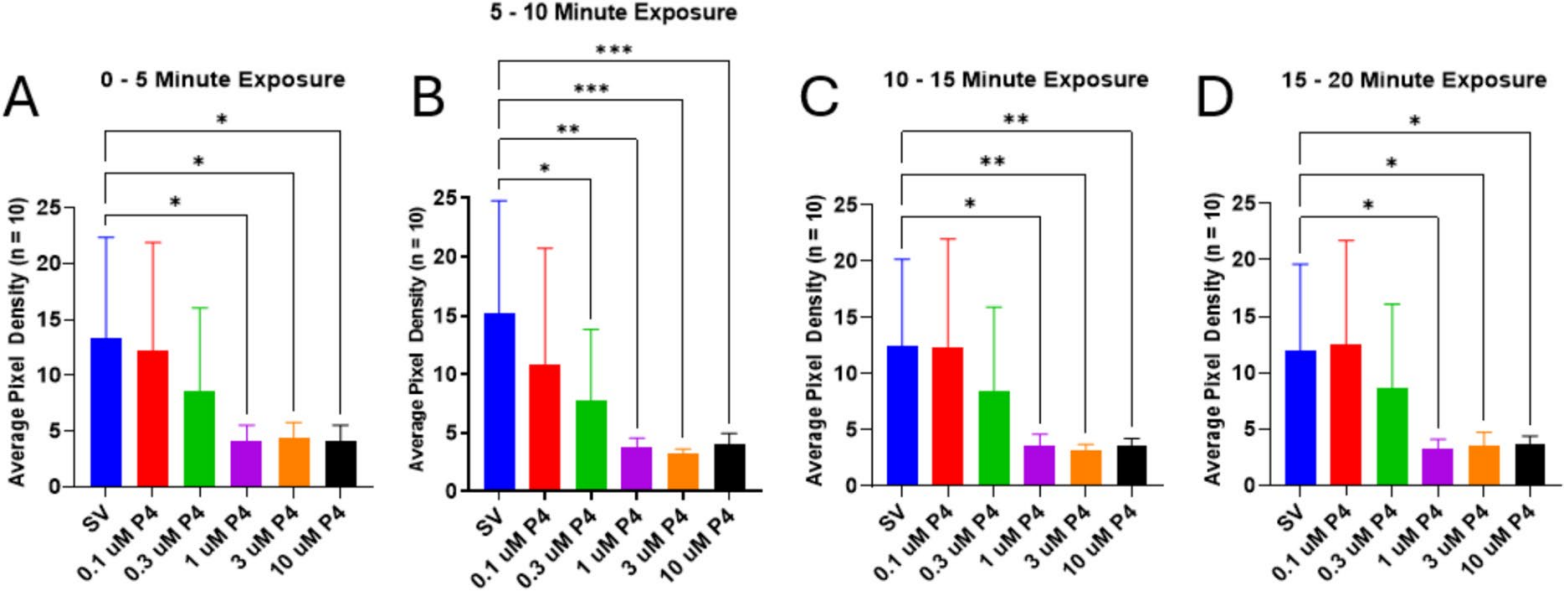
Locomotor activity of 7-dpf larval zebrafish decreases with increasing P4 concentration and over time. **A** Motility of P4-exposed larvae is significantly reduced within 5 min at 1 µM and higher concentrations of P4; **B** 5–10-min interval; **C** 10–15-min interval; **D** 15–20-min interval. *p < 0.05; **p < 0.01; ***p < 0.001. Values are mean ± SD and were analyzed by one-way ANOVA with Dunnett’s post hoc multiple-comparison test

To determine the concentrations of P4 that modulate locomotor behaviors, we measured swim behaviors in larval-stage zebrafish exposed to P4 in a dose-dependent manner (Fig. 2). Locomotor activity in larval zebrafish was quantified as APD of the fish within each well. Fish that have higher motility will be in various locations within the well every time an image is captured. When these images are stacked on top of each other, given a specified time interval, high motility will be depicted as what appears to be many zebrafish larvae in the well, whereas in fish that do not move, the image will show only one fish in the well. Therefore, we can measure the pixel density of the zebrafish within each well to get a measure of motility. High motility is represented by a higher APD, while fish that have lower motility show a lower APD.

The anesthetic effects of P4 in the 7-dpf larvae occur quickly within a matter of minutes (Fig. 2). Locomotor activity in zebrafish larvae exposed to 1, 3, and 10 µM P4 was significantly reduced within 5 min of exposure, as is represented by the decreasing APD of the zebrafish in each well with their respective treatments. There is a decreasing trend in locomotor activity as P4 concentration increases; however, larvae exposed to 0.1 and 0.3 µM showed no significant difference in locomotor activity compared to the control fish. Between 5 and 10 min, the average pixel density of the zebrafish exposed to 0.3 µM P4 decreases significantly; however, at later time points, the difference is no longer significant. At each time point after 10 min, exposure to 1, 3, and 10 µM P4 results in significantly decreased locomotor activity (Fig. 2).

**Fig. 3:**
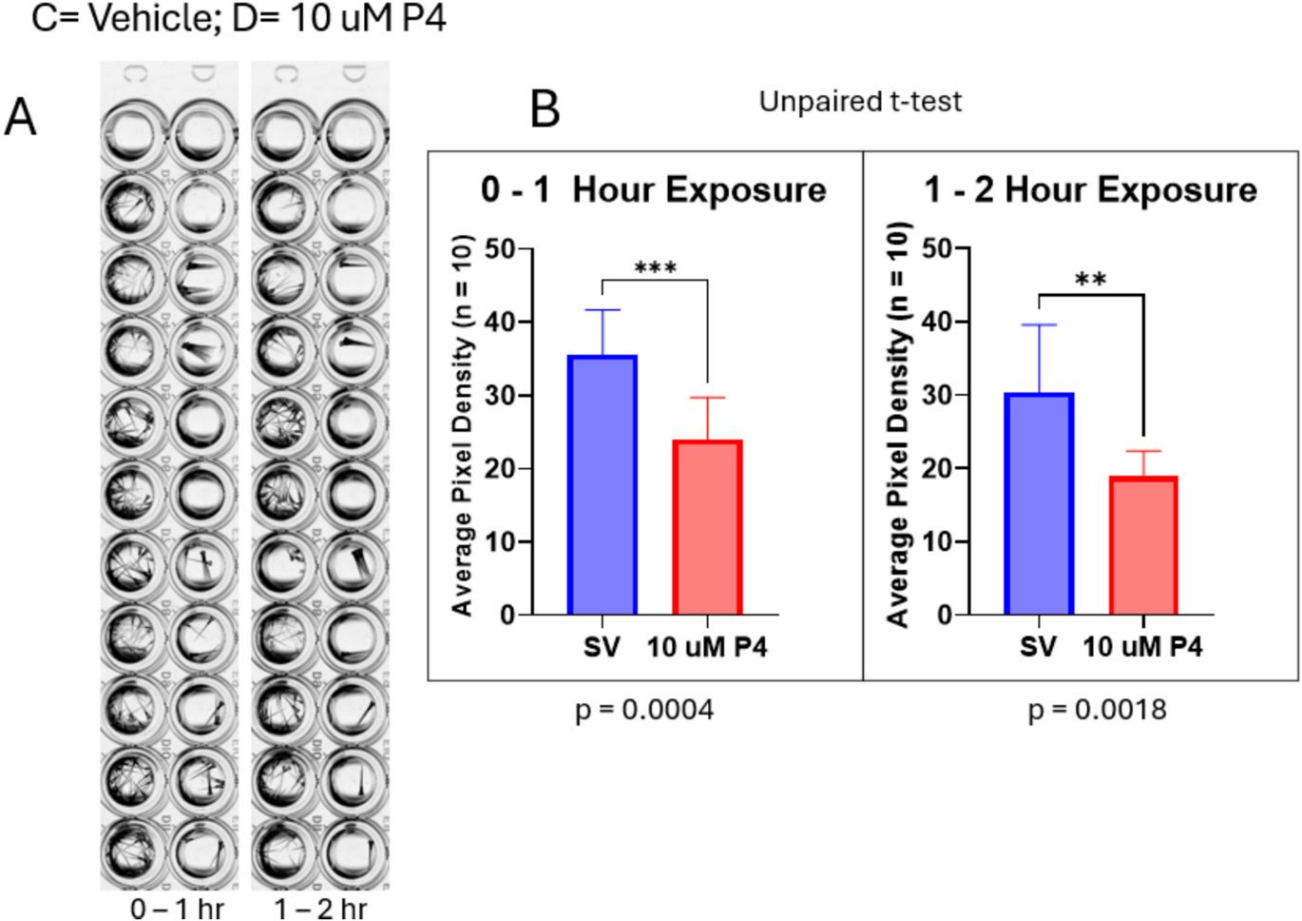
P4 at 10 µM markedly reduces motility without producing non-specific toxicity in 5-dpf zebrafish larvae. **A** Stacked images of larvae after vehicle and P4 exposure; **B** during the 1–2-h exposure period, motility is significantly decreased in the 10-µM P4 group (*p* < 0.01; *n* = 10). Values are mean ± SD and were analyzed with an unpaired *t* test

A motility rescue experiment to confirm that P4 was not having a non-specific, non-reversible toxic effect was performed. Images were captured of 5-day-old larvae incubated with 10 µM P4 for 1.5 h in a 96-well plate, then washed with untreated egg water prior to a further 2-h incubation with fresh untreated egg water. The locomotor activities of the larvae were then assessed for a second time. The images were stacked at 1-h intervals to assess locomotor effects before and after the wash. As observed previously, larvae treated with 10 µM P4 exhibited significant decreases in motility (Fig. 3).

**Fig. 4:**
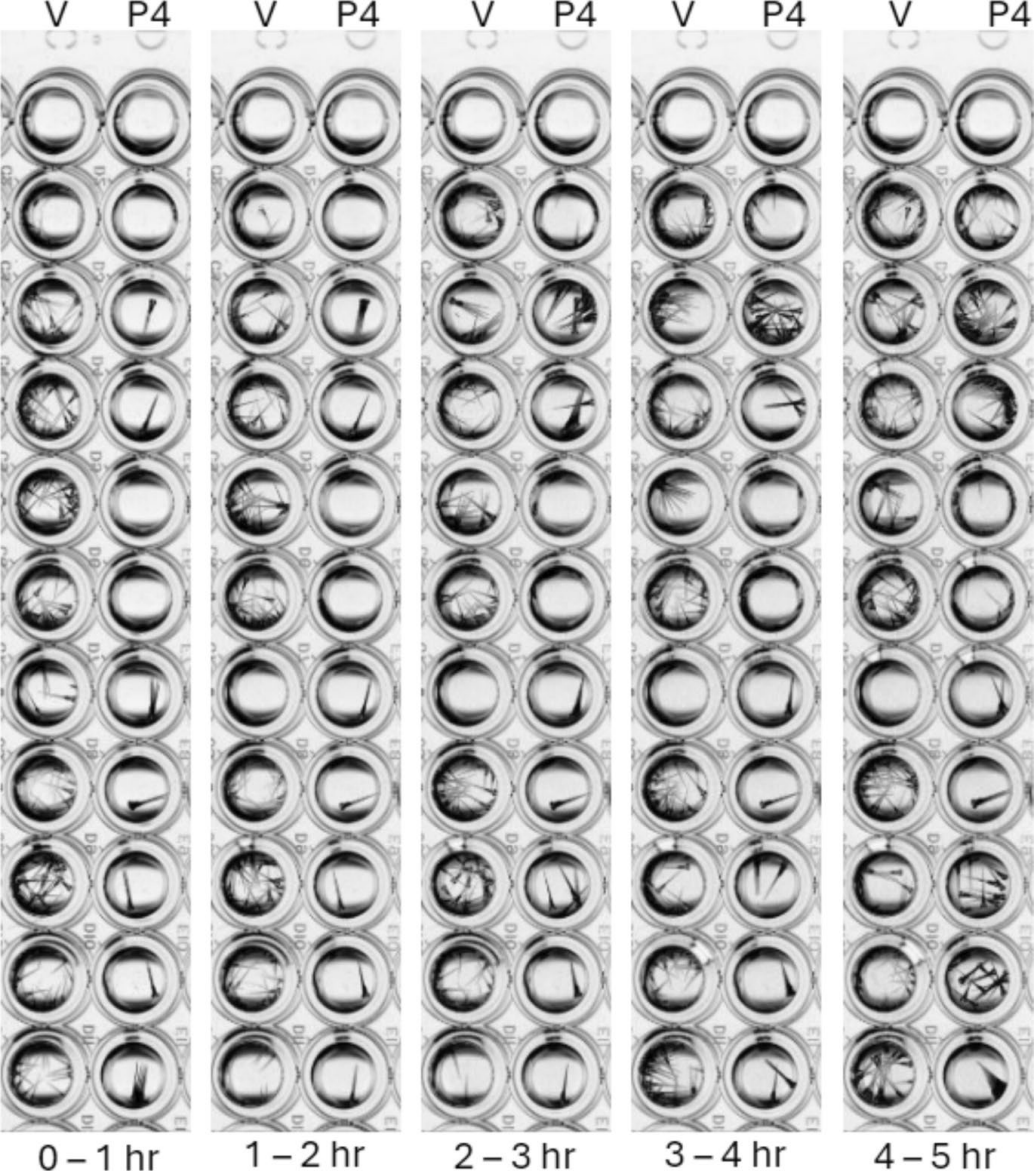
Images of 5-dpf zebrafish larvae after washing out P4 and replacing it with untreated egg water. *V*, steroid vehicle control; P4, 10 µM progesterone

**Fig. 5:**
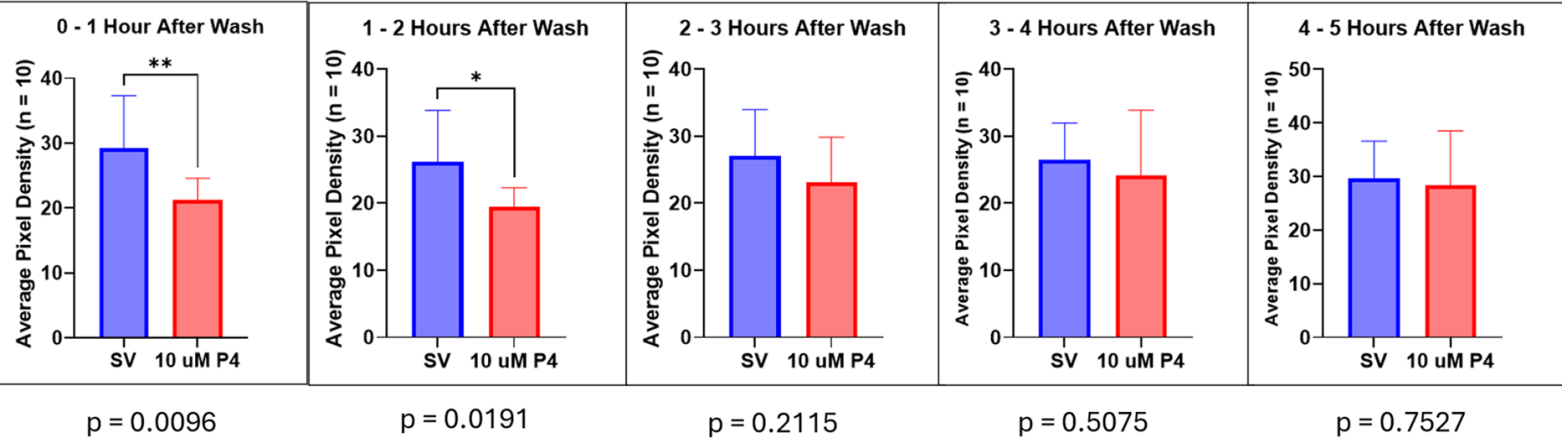
P4 at this concentration and exposure duration does not cause non-specific toxicity in 5-dpf zebrafish larvae. After 2 h of washout, locomotor activity recovers and is not significantly different from control levels (*p* > 0.05; *n* = 10). Values are mean ± SD and were analyzed with an unpaired *t* test

Upon washing out the P4 and placing the larvae back in clean egg water, motility was recovered after 2 h and was no longer significantly different from control levels (*p* > 0.05) (Figs. 4 and 5). Progesterone was not toxic at the concentrations tested, which caused significant decreases in motility, with this effect lasting for more than 1 h and reversing after drug washout (Fig. 5).

### Embryonic stage zebrafish (24 hpf) are insensitive to the P4-induced inhibition of locomotor activity and instead exhibit increased motility

**Fig. 6:**
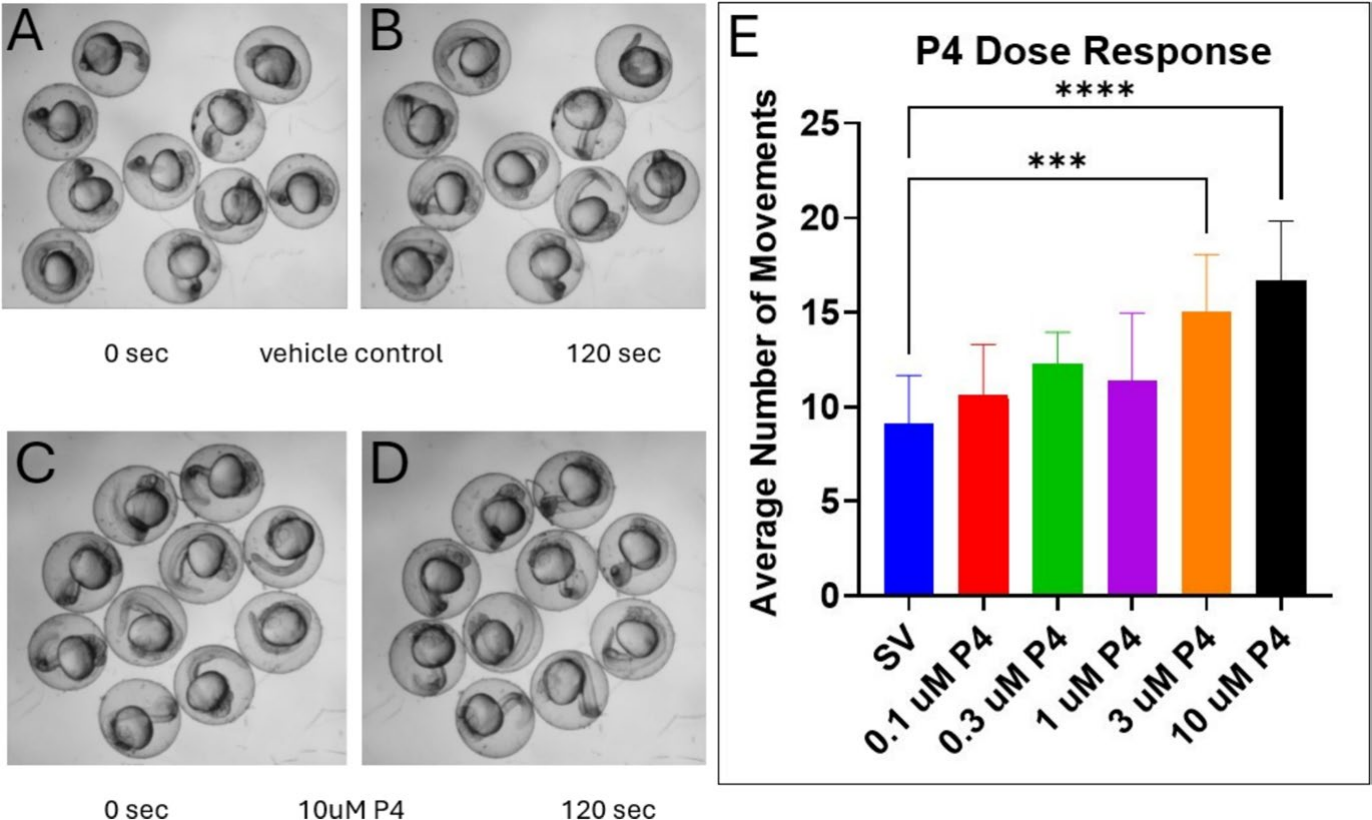
Zebrafish larvae (24 hpf) exhibit increased spontaneous tail coiling when exposed to rising concentrations of P4. **A**, **B** Images of 24-hpf zebrafish embryos exposed to steroid vehicle control; **C**, **D** images of 24-hpf embryos exposed to 10 µM P4 at 0 and 120 s, showing the different embryo orientations within the chorion due to spontaneous tail coiling. **E** After 1.5 h of exposure, spontaneous tail coiling significantly increased in embryos treated with 3 and 10 µM P4 compared to controls (***p < 0.001; ****p < 0.0001; *n* = 10). Values are mean ± SD and were analyzed by one-way ANOVA with Dunnett’s post hoc multiple-comparison test

Motor behaviors in developing zebrafish appear in a specific sequential manner and consist of an early period of transient spontaneous tail coiling behaviors starting around 17 hpf, followed by touch-induced twitch and swim responses by 27 hpf, then stereotypical swimming patterns emerge at 5 dpf (Saint-Amant and Drapeau 1998; Drapeau et al. 2002; Brustein et al., 2003). We measured the anesthetic effect of P4 on embryonic-stage zebrafish by exposing the embryos to increasing concentrations of P4 for 1.5 h before recording 2-min videos of the 24-hpf embryos and counting how many tail contractions the embryos exhibited (Fig. 6A, B) under steroid vehicle treatment and under 10 µM P4 treatment (Fig. 6C, D). Spontaneous coiling behaviors exhibited by the embryos had a dose-dependent increasing trend as P4 concentrations increased (Fig. 6E). At 3 and 10 µM P4, the average number of spontaneous tail coiling across 10 embryos per treatment significantly increased compared to the control.

### Nuclear steroid receptors (nPR and GR) are unlikely to mediate changes in locomotor activity

**Fig. 7:**
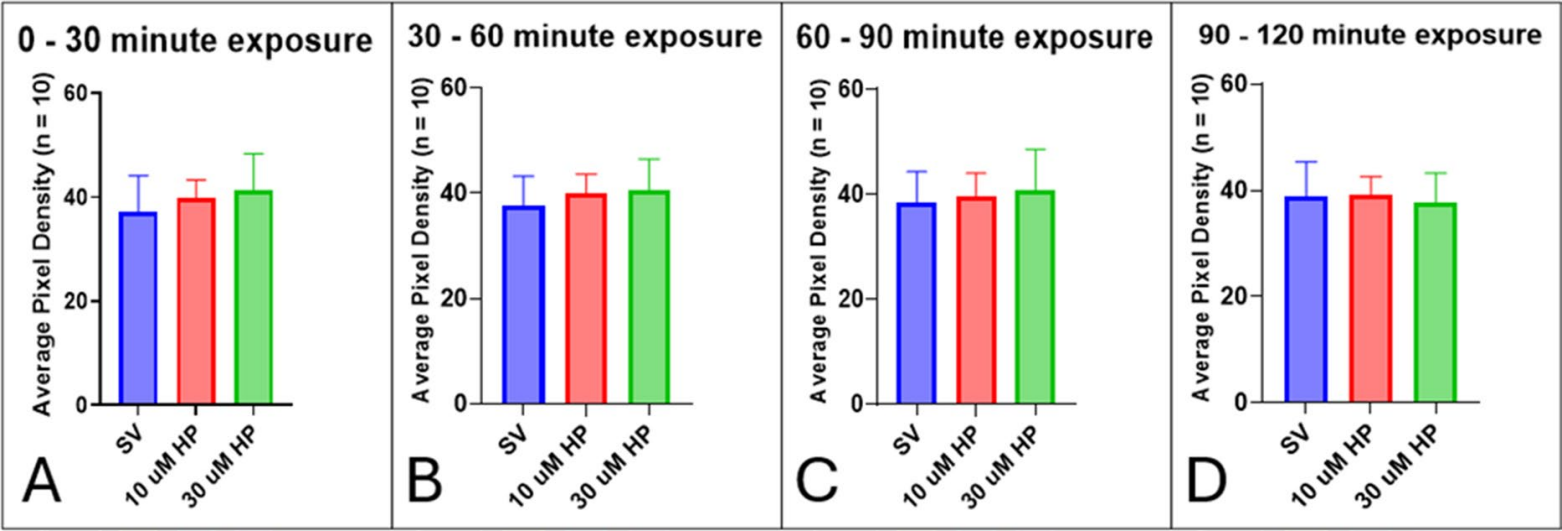
Motility of 7-dpf zebrafish larvae exposed to 10 and 30 µM 17α-hydroxyprogesterone (HP), recorded in 30-min intervals: **A** 0–30 min, **B** 30–60 min, **C** 60–90 min, and **D** 90–120 min; *p* > 0.05

P4 can act on different receptors (Table 1) to facilitate its biological effects. P4 is a neurosteroid and can alter behavior, while one of its metabolites, 17α-HP, acts as a natural ligand on the nPR and is not reported to have behavioral effects (Diotel et al. 2011). To assess the role of the nPR in affecting locomotor behaviors in larval and embryonic zebrafish, 17α-HP and mifepristone (RU486), an inhibitor of the nPR, were utilized in co-exposure experiments. The locomotor activity of 7-dpf larval zebrafish was not significantly altered across all time intervals when exposed to both concentrations of 17α-HP (Fig. 7). After a 2-h exposure, locomotor activity was similar in fish exposed to steroid vehicle and 10 and 30 µM 17α-HP (control vs. 10 µM 17α-HP, *p* = 0.99; control vs. 30 µM 17α-HP, *p* = 0.89). Similarly, motility was not significantly altered between both concentrations of 17α-HP (10 vs. 30 µM 17α-HP, *p* = 0.84).

**Fig. 8:**
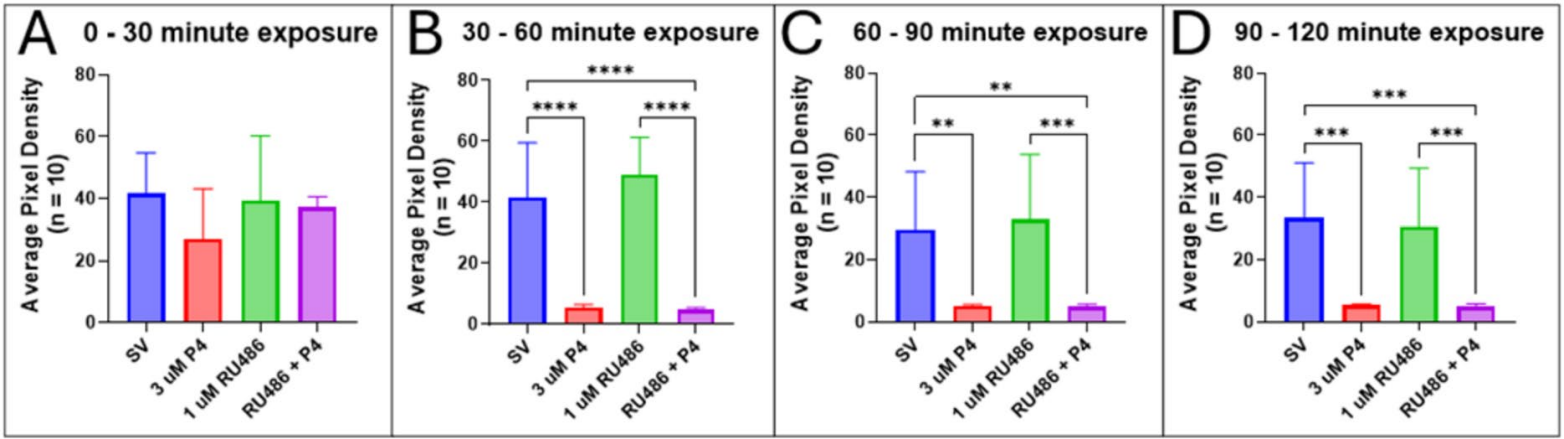
Inhibition of nPR by RU486 does not prevent P4-induced decrease in locomotor activity in 7-dpf zebrafish larvae. Values are mean ± SD and were analyzed by one-way ANOVA with Tukey’s post hoc multiple-comparison test

Larvae incubated with RU486 (1 to 10 µM) did not show any signs of altered locomotor behavior across all time points up to 4 h after exposure. These results show that a 30-min pre-treatment incubation with 1 µM RU486 in 7-dpf zebrafish did not prevent the anesthetic effect induced by subsequent exposure to 3 µM P4 (Fig. 8). Between 0 and 30 min, there is no significant difference in motility exhibited by larvae exposed to the control, RU486, P4, and a combination of both (Fig. 8A). After 30 min of P4 exposure, for larvae pre-treated with RU486 or not, motility is significantly diminished compared to the control and RU486 alone groups (Fig. 8B; SV control vs. 3 µM P4, *p* < 0.0001, 1 µM RU486 vs. RU486 + P4, *p* < 0.0001, SV control vs. RU486 + P4, *p* < 0.0001). These significant differences in motility are reflected in later time periods (Fig. 8C, D).

**Fig. 9:**
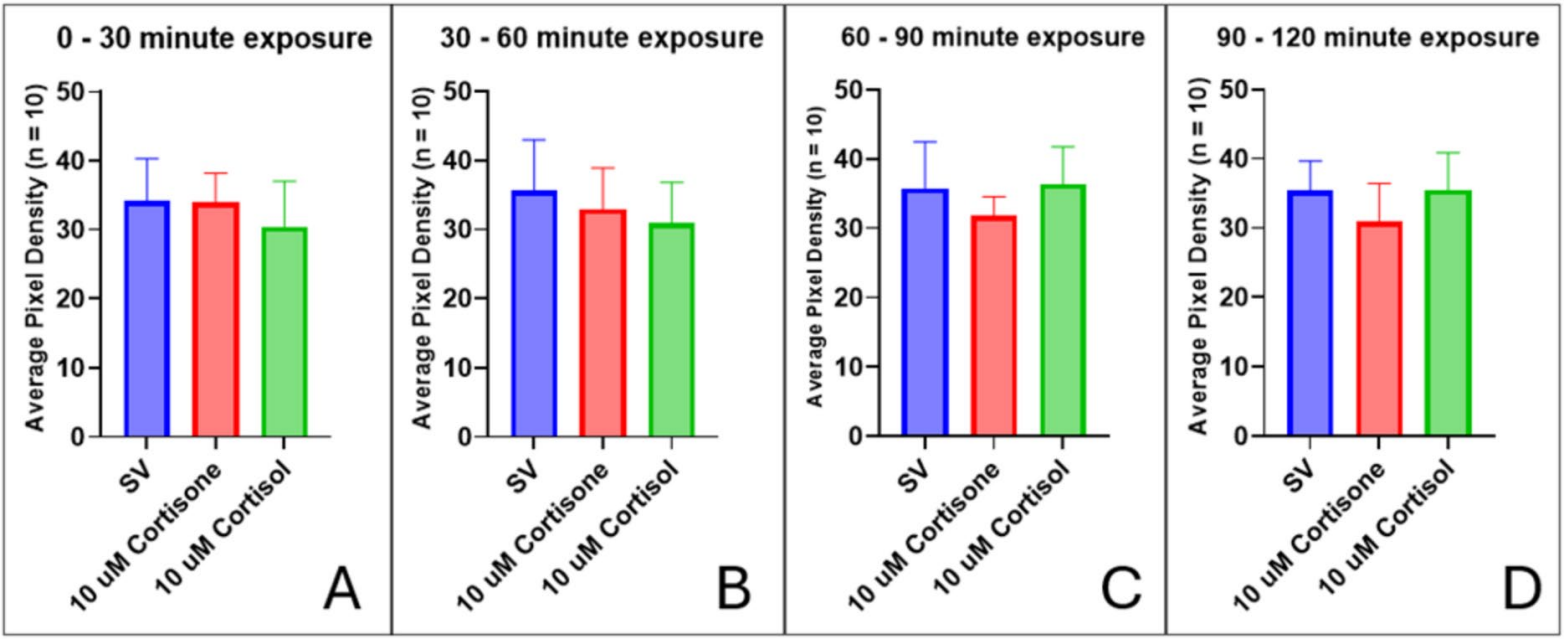
Activation of the GR using cortisol and cortisone has no significant effect on locomotor activity in 7-dpf zebrafish larvae. Motility was recorded in 30-min intervals: **A** 0–30 min, **B** 30–60 min, **C** 60–90 min, and **D** 90–120 min. Values are mean ± SD and were analyzed by one-way ANOVA with Tukey’s post hoc multiple-comparison test

**Fig. 10:**
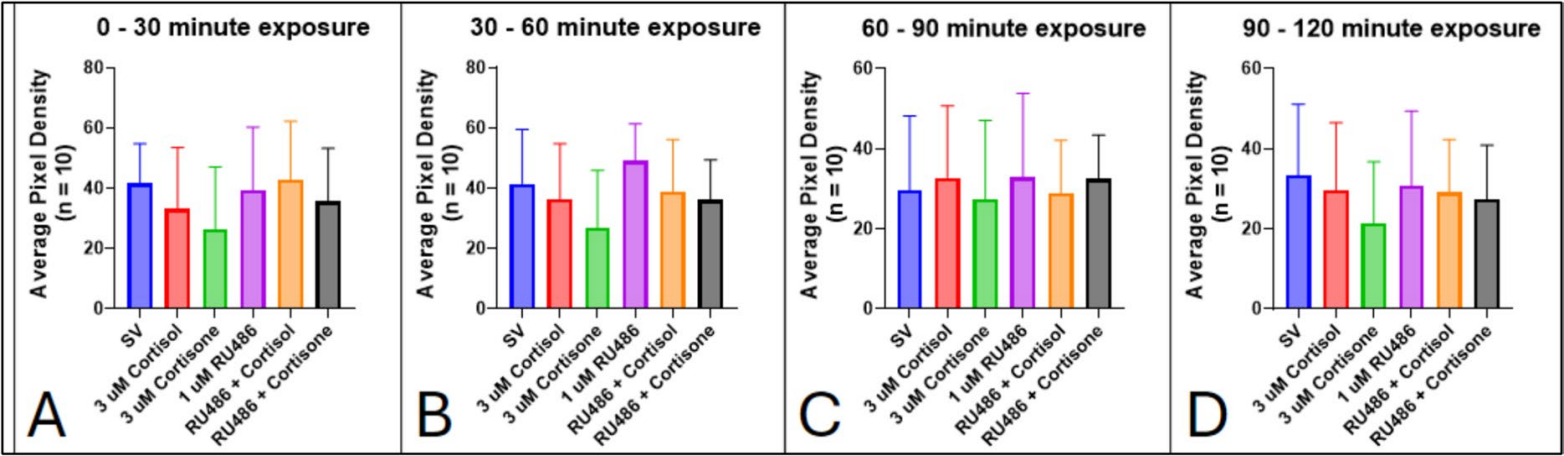
Inhibition of the GR with RU486 does not significantly alter locomotor activity in 7-dpf zebrafish. Motility was measured in 30-min intervals: **A** 0–30 min, **B** 30–60 min, **C** 60–90 min, and **D** 90–120 min. Values are mean ± SD and were analyzed by one-way ANOVA with Tukey’s post hoc multiple-comparison test

Progesterone is a weak agonist of the GR; therefore, we utilized the glucocorticoids cortisol and cortisone to test whether activation of the GR affects motility. Zebrafish larvae (7 dpf) motility did not change by 2 h after exposure to cortisol and cortisone (Fig. 9). Locomotor activity remained comparable to the levels measured in steroid vehicle control fish (SV vs. cortisol, *p* = 0.9984; SV vs. cortisone, *p* = 0.1116) (Fig. 9D). Also, the nPR antagonist RU486 may have partial antagonistic activity at the GR (Table 1). To assess whether locomotor activity may be modulated through activation of the GR, larvae were pre-treated with RU486 and then exposed to cortisol or cortisone. Exposure to RU486 alone did not alter locomotor activity compared to the control by 2 h (SV vs. RU486, *p* = 0.9991) (Fig. 10D). When larvae were pre-treated with RU486 and then subsequently exposed to cortisol or cortisone, locomotor activity was unaffected and remained comparable to SV controls (SV vs. RU486 + cortisol, *p* = 0.9911; SV vs. RU486 + cortisone, *p* = 0.9610) (Fig. 10D). To assess whether the application of RU486 was spilling over to interact with the GR, we pre-treated 7-dpf larvae with 1 µM RU486 for 30 min before adding 3 µM cortisol or cortisone (Fig. 10). There was no significant change in locomotor activity in all treatment groups.

### Inhibition of PGRMC1 using AG205 and its effect on locomotor activity

**Fig. 11:**
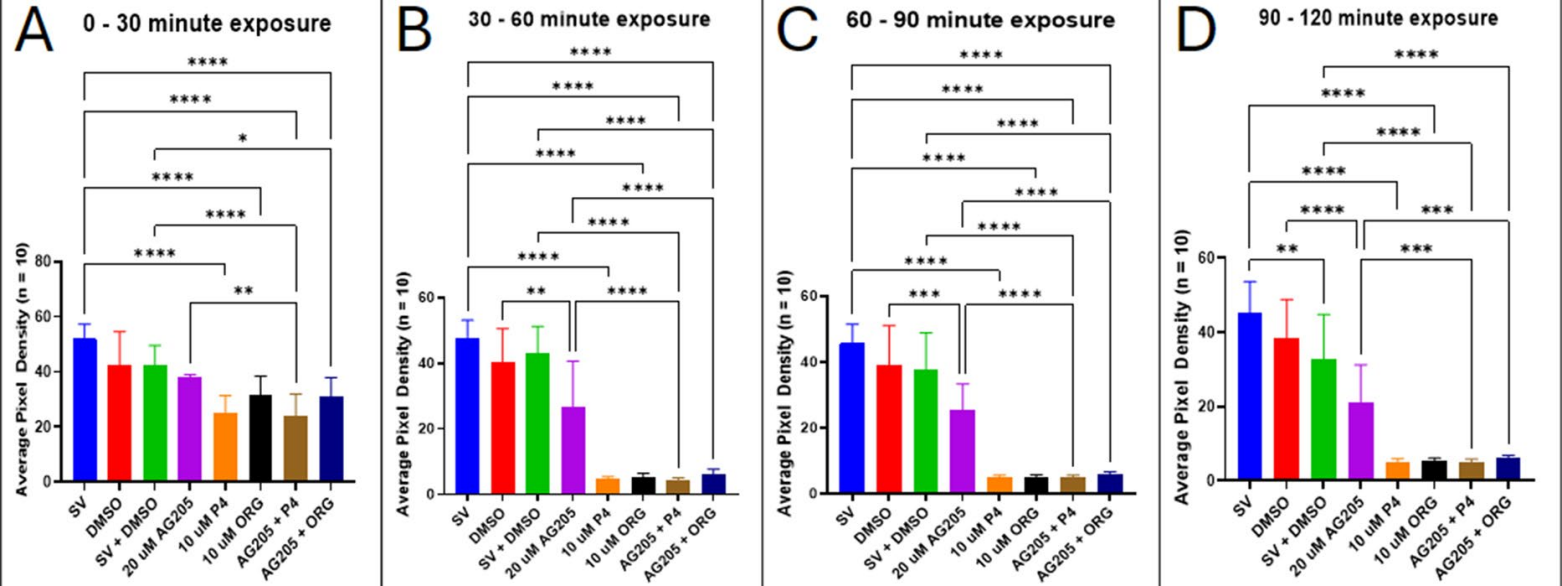
Inhibition of PGRMC1 using AG205 does not affect locomotor activity in 5-dpf zebrafish. Motility was recorded over successive 30-min intervals: **A** 0–30 min, **B** 30–60 min, **C** 60–90 min, and **D** 90–120 min. *p < 0.05; **p < 0.01; ***p < 0.001; ****p < 0.0001; *n* = 10

Progesterone receptor membrane component 1 (PGRMC1) is a single-pass transmembrane heme-binding protein that is part of the 335 membrane-associated progesterone receptor (MAPR) family (Cahill, 2007; Kimura et al., 2012). To test the role that PGRMC1 may play in modulating locomotor behaviors, 5-dpf zebrafish larvae were pre-treated with the PGRMC1 inhibitor, AG205 (Table 1), then exposed to P4 or ORG, a specific mPR agonist. Inhibiting PGRMC1 with AG205 did not prevent P4- or ORG-induced reductions in locomotor behaviors compared to the controls (*p* < 0.0001). However, there was also a significant reduction in motility in larvae exposed to 20 µM AG205 compared to the DMSO control (DMSO vs. AG205, *p* = 0.0011) (Fig. 11).

### Selective activation of the membrane progesterone receptor

**Fig. 12:**
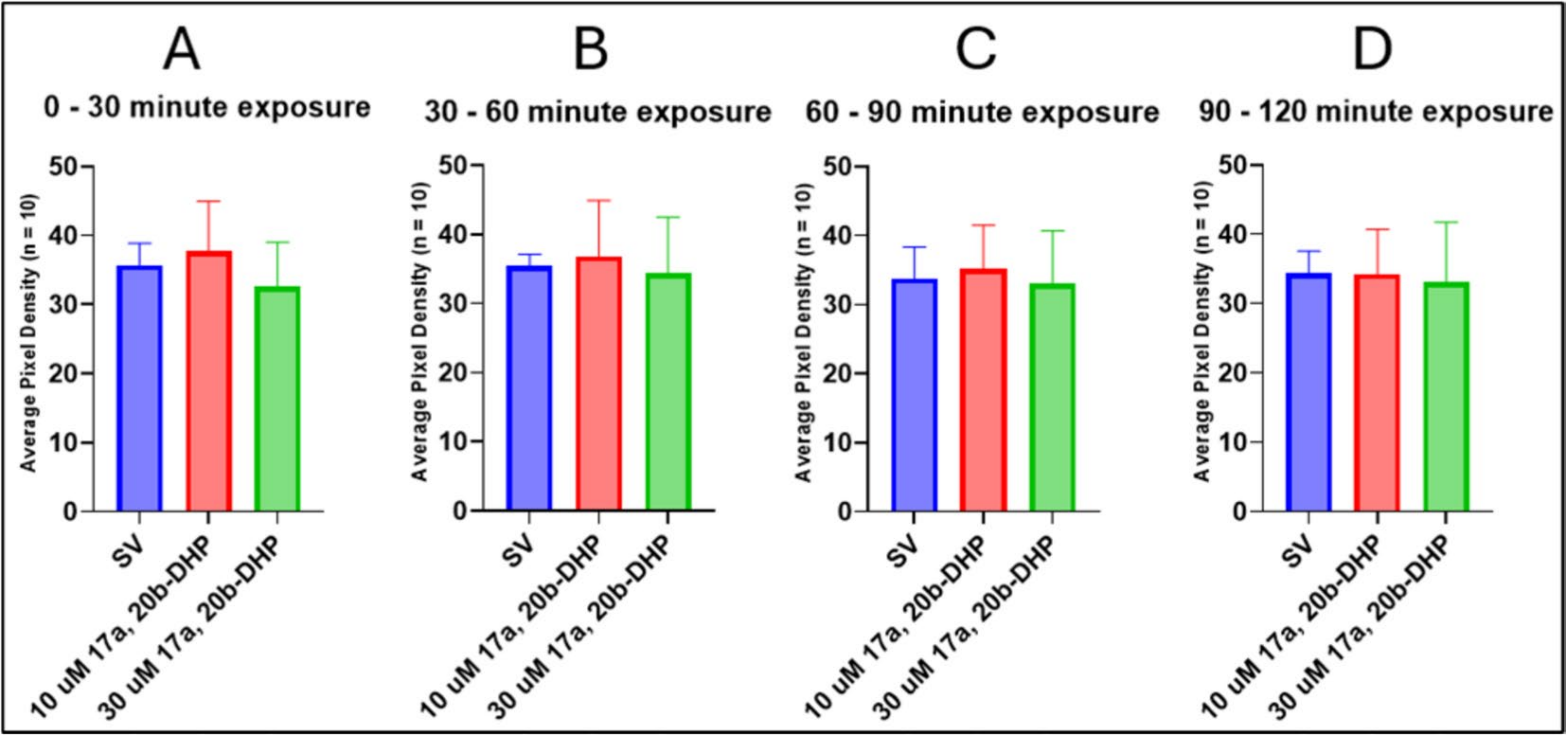
Exposure to the natural mPR agonist 17α,20β-DHP does not alter locomotor activity in 7-dpf zebrafish. Motility was measured during consecutive intervals: **A** 0 and 30 min, **B** 30 and 60 min, **C** 60 and 90 min, and **D** 90 and 120 min after exposure

To evaluate the role that mPR plays in locomotor inhibition, 7-dpf larvae were exposed to 17α,20β-DHP, which is the natural steroid hormone in zebrafish that promotes oocyte maturation by activating the mPR found on the surface of the oocyte, or a selective mPR agonist, ORG. Exposure to both 10 and 30 µM 17α,20β-DHP did not significantly alter locomotor activity in larval zebrafish compared to the steroid vehicle controls at all time intervals measured (SV vs. 10 µM 17α,20β-DHP, *p* = 0.8360; SV vs. 30 µM 17α,20β-DHP, *p* = 0.9200) (Fig. 12).

**Fig. 13:**
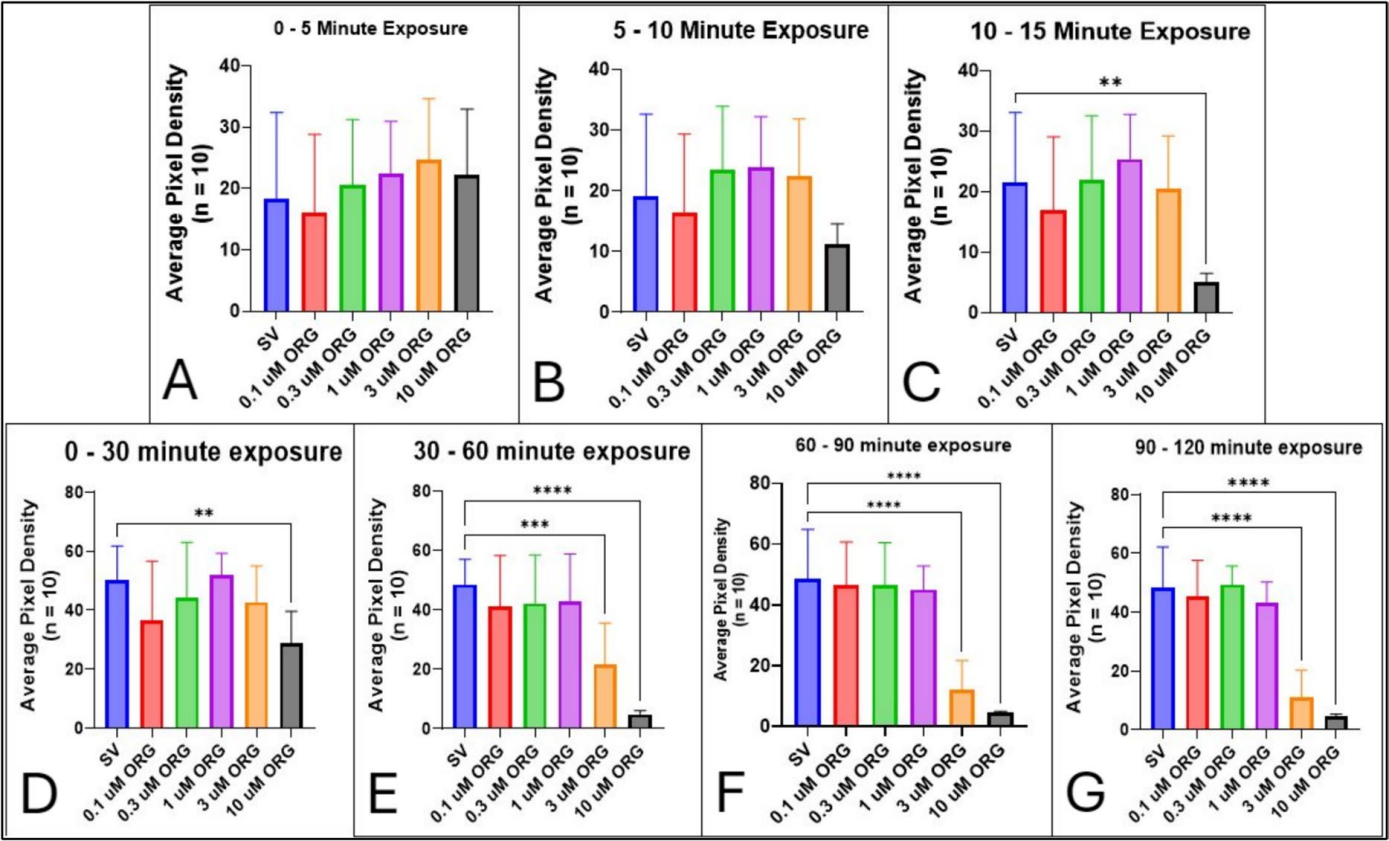
Exposure to the selective mPR agonist ORG-OD-02–0 significantly reduces locomotor activity in 5-dpf zebrafish. **A** ORG at 10 µM suppresses motility within the first 30 min. **B** Concentrations of 3 µM ORG and higher elicit an anesthetic-like effect, evidenced by markedly lower locomotor activity after 30 min, whereas ≤ 1 µM have no such effect even after 2 h of exposure. *N* = 10 per treatment; **p < 0.01; ***p < 0.001; ****p < 0.0001. Values are mean ± SD and were analyzed by one-way ANOVA with Dunnett’s post hoc multiple-comparison test

In contrast with 17α,20β-DHP, treatment with ORG caused significant decreases in motility (Fig. 13). There was a trend in the decrease of locomotor activity in 5 dpf larvae by 10 µM ORG that occurred within 10 min of exposure (SV vs. 10 µM ORG, *p* = 0.296) (Fig. 13B), although significant inhibition did not occur until between 10 and 15 min (SV vs. 10 µM ORG, *p* = 0.0012) (Fig. 13C). Exposure to 3 µM ORG also induced a statistically significant reduction in locomotor behavior; however, this occurred after 30 min of exposure (SV vs. 3 µM ORG, *p* = 0.0002) (Fig. 13D). Concentrations of ORG below 3 µM did not significantly alter motility in zebrafish larvae, even up to 2 h of exposure (SV vs. 1 µM ORG, *p* = 0.598) (Fig. 13G). Additionally, the IC_50_ of ORG affecting locomotor activity was determined through a dose–response assay and using a nonlinear curve fitting of the responses of the concentrations applied. IC_50_ values were calculated for each 30-min time interval. The IC_50_ of ORG was 1.66 µM (Table 2) between 0 and 30 min, 7.42 µM between 30 and 60 min, 7.24 µM between 60 and 90 min, and 3.26 µM between 90 and 120 min after exposure (Table 2).

**Table 2:** Summary of IC_50_ values for the effect of each steroid on locomotor activity in 5–7-dpf zebrafish larvae, shown in 30-min intervals

Steroid	0–30 min	30–60 min	60–90 min	90–120 min
P4 (7 dpf)	0.36 µM	0.7 µM	0.93 µM	1.15 µM
ORG (5 dpf)	1.66 M	7.419 µM	3.585 µM	3.256 µM
DOC (5 dpf)	0.7 µM	1.73 µM	1.692 µM	3.3 µM
5α-DHP (5 dpf)	0.549 µM	0.342 µM	0.351 µM	0.33 µM

### P4 and its metabolites may act on the GABA-A receptor as a positive allosteric modulator

**Fig. 14:**
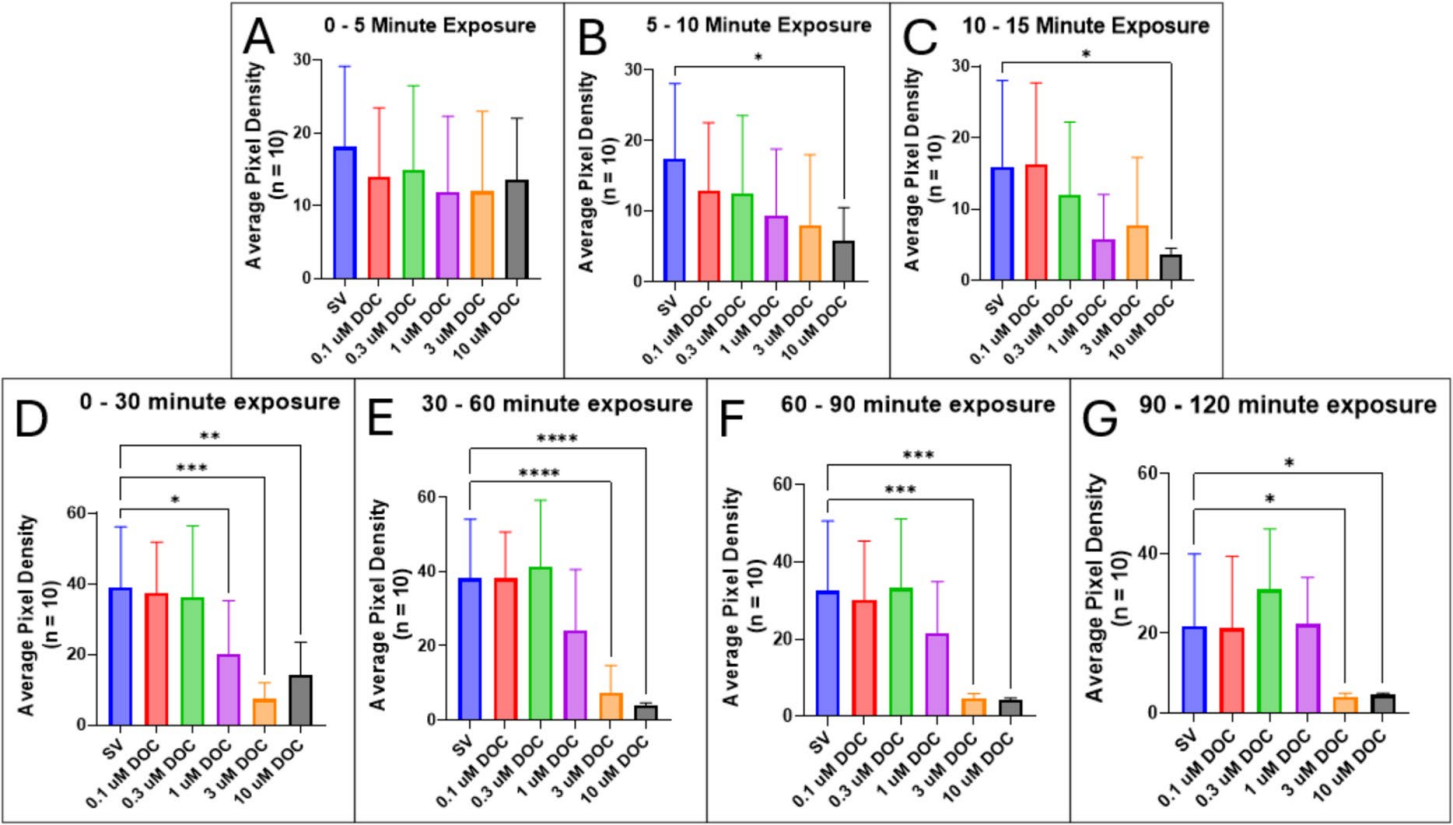
Locomotor activity of 5-dpf zebrafish larvae (*n* = 10) is significantly reduced by DOC. Motility was recorded in successive intervals: **A** 0–5 min, **B** 5–10 min, **C** 10–15 min, **D** 0 and 30 min, **E** 30 and 60 min, **F** 60 and 90 min, and **G** 90 and 120 min after DOC exposure. Values are mean ± SD and were analyzed by one-way ANOVA with Dunnett’s post hoc multiple-comparison test. *p < 0.05; **p < 0.01; ***p < 0.001; ****p < 0.0001

DOC is a mineralocorticoid that is derived from progesterone. It is also the precursor to THDOC, which is a known PAM of the GABA_A_ receptor. Results from motility assays show that larvae acutely exposed to 10 µM DOC exhibited a significant reduction in motility after 5 min of exposure compared to those exposed to the steroid vehicle control (SV vs. 10 µM DOC, *p* = 0.041), although lower concentrations do not induce significant changes in motility at this time point (Fig. 14B).

After 30 min of exposure, locomotor behaviors are significantly attenuated in larvae exposed to 1, 3, and 10 µM compared to the steroid vehicle-exposed control group (SV vs. 1 µM DOC, *p* = 0.0143; SV vs. 3 µM DOC, *p* = 0.0065; SV vs. 10 µM, *p* = 0.0059) (Fig. 14D). However, lower doses of 0.1 and 0.3 µM did not significantly alter locomotor behaviors. After 30 min and up to 2 h after exposure to DOC, only doses of 3 and 10 µM induce significant decreases in motility (Fig. 14E–G), while larvae exposed to 1 µM DOC showed no significant difference in motility compared to the control. A dose–response assay shows that the IC_50_ of DOC exposure to reduce locomotor activity after 1 h is 1.734 µM (Table 2).

Similar to P4, ORG, and DOC, 5-dpf zebrafish larvae incubated with the direct precursor to allopregnanolone, 5α-DHP, showed significantly reduced locomotor behaviors.

**Fig. 15:**
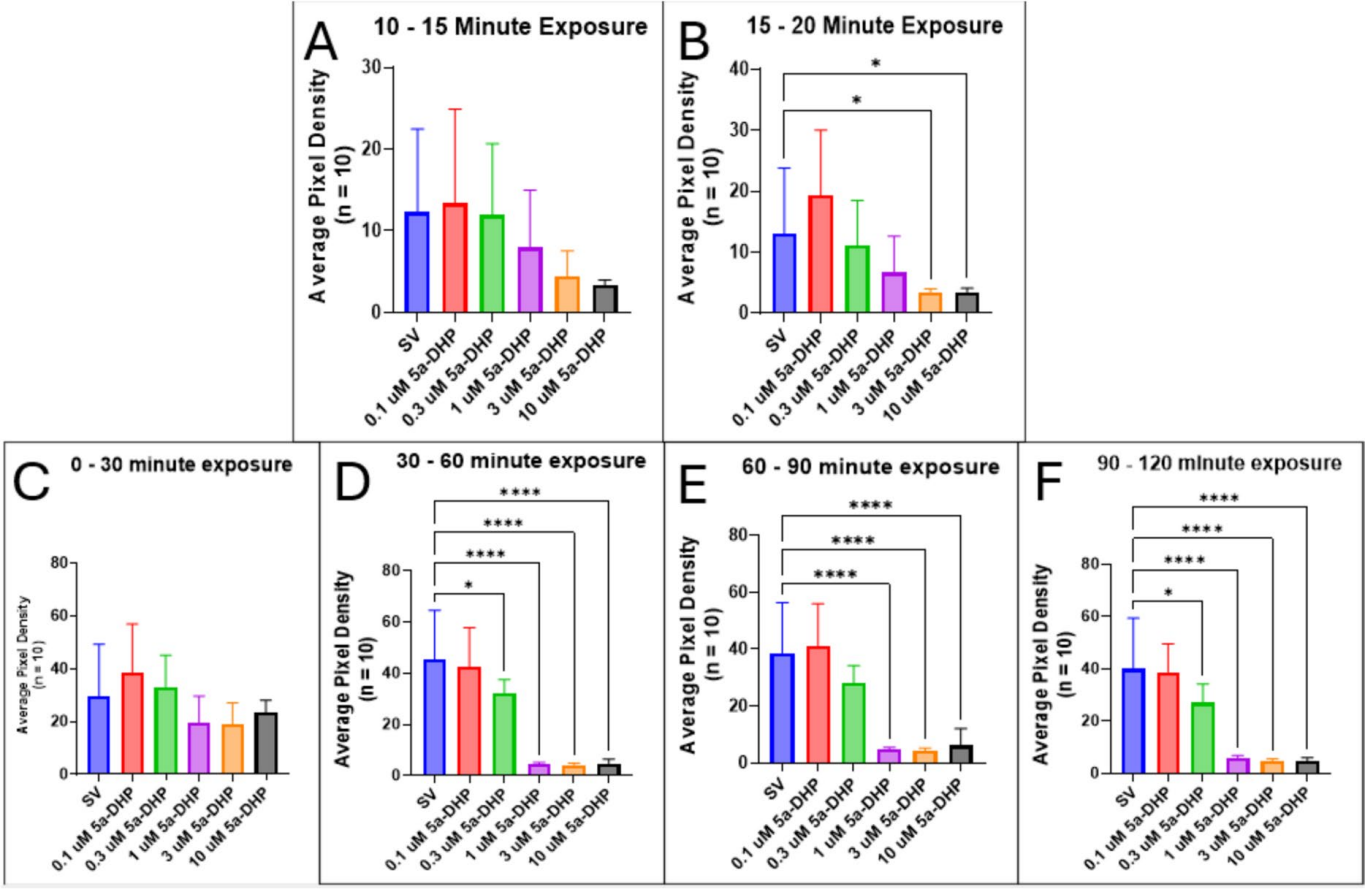
Effect of 5α-DHP on locomotor activity in 5-dpf zebrafish larvae. **A** Locomotor activity decreases after 10 min of exposure. **B** This decrease is not significant until after 15 min of exposure to 5α-DHP. **C** No significant difference is observed during the first 0 and 30 min. **D** By 60 min, motility is significantly lower in larvae exposed to 0.3 to 10 µM 5α-DHP. **E** Motility is decreased in the 1-, 3-, and 10-µM treatment groups during 60 and 90 min. **F** Motility between 90 and 120 min. Values are mean ± SD and were analyzed by one-way ANOVA with Dunnett’s post hoc multiple-comparison test, *n* = 10. *p < 0.05; ****p < 0.0001

After 60 min of exposure, motility is significantly reduced at concentrations ≥ 0.3 µM (SV vs. 0.3 µM 5α-DHP, *p* = 0.0249; SV vs. 1, 3, 10 µM 5α-DHP, *p* < 0.0001), whereas the lowest concentration tested does not differ from the vehicle control (Fig. 15D). This pattern is also observed 90 min after exposure to 5α-DHP, with the exception of the 0.3 µM treatment group, which is not significantly different compared to the control group (Fig. 15E). By 120 min after initial exposure, 0.3 µM induces significantly lower locomotor behaviors compared to the control (Fig. 15F). A dose–response curve of the anesthetic effect of 5α-DHP exposure on motility revealed that between 0 and 30 min of exposure, the IC_50_ is 0.549 µM (Fig. 21A; Table 2). Between 30 and 60 min of exposure, the IC_50_ for inhibited motility is 0.342 µM (Table 2). The value is 0.351 µM at 60–90 min and 0.33 µM at 90–120 min (Table 2).

**Fig. 16:**
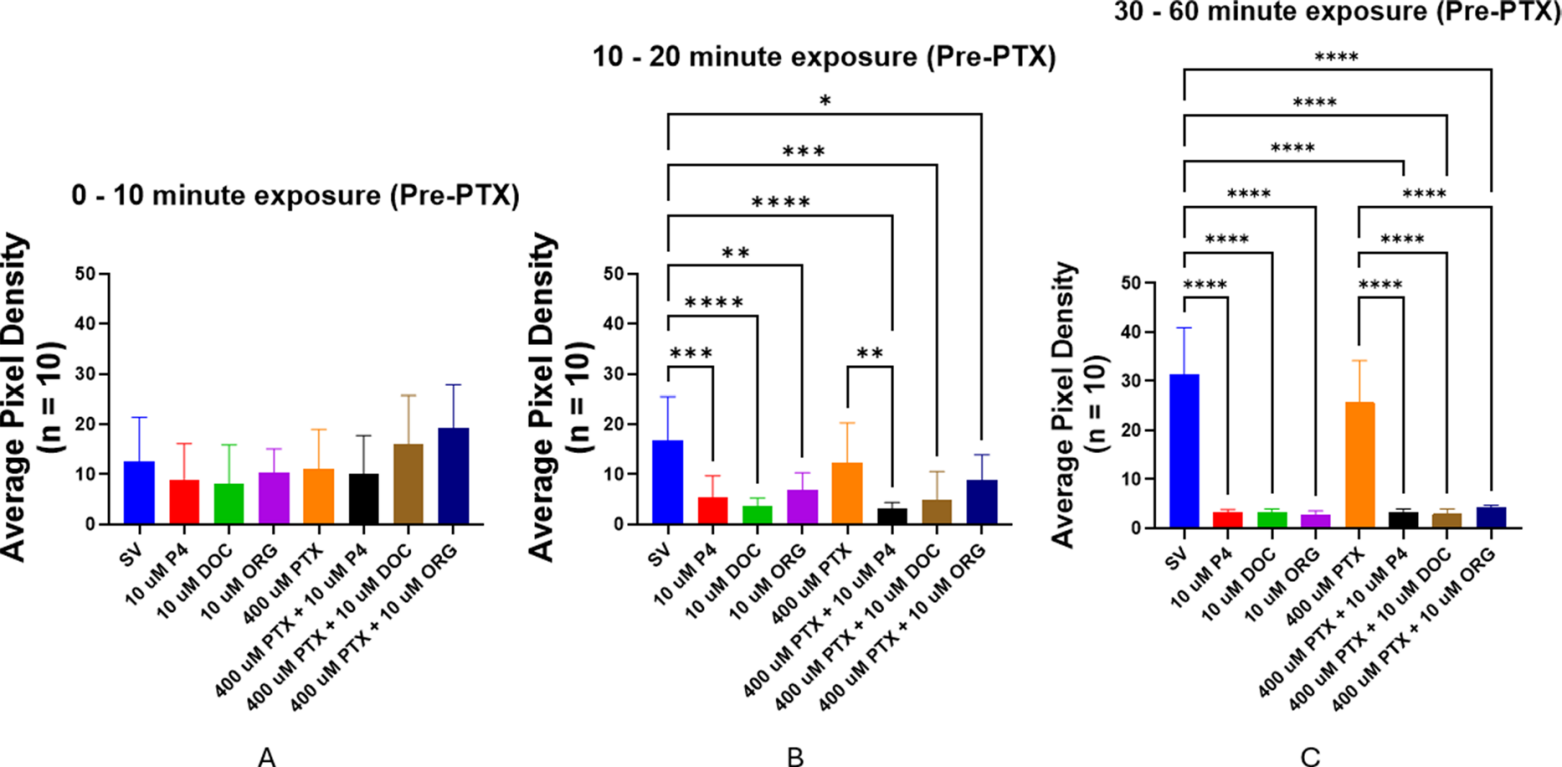
Locomotor activity (expressed as average pixel density of stacked images) of 5-dpf zebrafish larvae *following* neurosteroid exposure but *before* PTX addition: **A** no significant change during 0 and 10 min; **B** motility is significantly reduced at 10 and 20 min; **C** activity measured between 30 and 60 min after exposure. Values are mean ± SD and were analyzed by one-way ANOVA with Tukey’s post hoc multiple-comparison test. *p < 0.05; **p < 0.01; ***p < 0.001; ****p < 0.0001

PTX is a compound that inhibits the GABA_A_ receptor by blocking the ion channel to prevent chloride (Cl^−^) ion influx. We used PTX in cotreatment locomotor activity assays to evaluate whether seizurogenic activity can be generated in larvae pre-treated with pregnane neurosteroids. Pre-treatment with 10 µM P4, DOC, or ORG resulted in expected significant decreases in APD, indicating a decrease in locomotor activity beginning after 10 min of exposure (SV vs. all 10 µM P4, *p* = 0.0003; SV vs. 10 µM DOC, *p* < 0.0001; SV vs. 10 µM ORG, *p* = 0.0023) (Fig. 16B). After a longer incubation period of up to 60 min, motility in all treatments containing neurosteroids was significantly reduced compared to the steroid vehicle (SV all neurosteroids, *p* < 0.0001) (Fig. 16C). It is worth noting that the PTX-only group was not exposed to PTX at this point, and larvae were incubated in clean egg water. This group of larvae had significantly higher motility than the cotreatment groups in which larvae were exposed to neurosteroids (Fig. 16B, C). Additionally, before PTX application, there was no significant difference between the SV control fish and the PTX group at any time interval (Fig. 16).

**Fig. 17:**
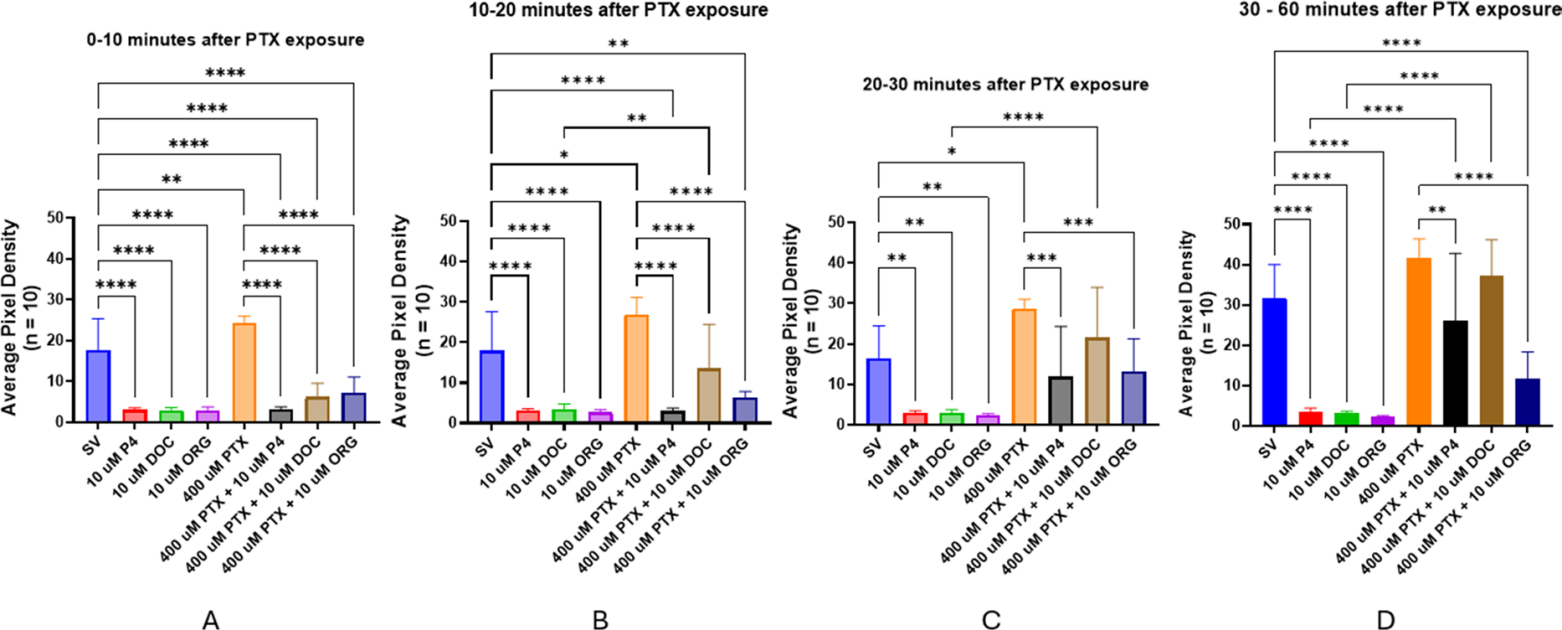
Effect of picrotoxin on motility in 5-dpf zebrafish larvae after a 60-min neurosteroid pre-treatment. Titles correspond to time intervals following PTX exposure, occurring after the 1-h neurosteroid pre-treatment period: **A** between 0 and 10 min; **B** between 10 and 20 min; **C** between 20 and 30 min; **D** between 30 and 60 min. Values are mean ± SD and were analyzed by one-way ANOVA with Tukey’s post hoc multiple-comparison test. *p < 0.05; **p < 0.01; ***p < 0.001; ****p < 0.0001

After 60 min of pre-treatment with steroids, 400 µM PTX was added to the respective cotreatment groups (PTX + neurosteroid). After PTX application, the PTX group caused a significant increase in locomotor activity (SV vs. 400 µM PTX, *p* = 0.0017) (Fig. 17A). After 10 min of PTX exposure (1 h and 10 min after neurosteroid exposure), larvae pre-treated with DOC exhibited a significant increase in motility compared to the DOC-alone (without PTX) (DOC vs. PTX + DOC, *p* < 0.0036) and were not different from the control group (Fig. 17A). Motility in larvae pre-treated with DOC is recovered 20 min after PTX application and is similar to the PTX-alone treatment group (PTX vs. PTX + DOC, *p* = 0.4219) (Fig. 17C). During the same time interval, motility of larvae pre-treated with P4 and then exposed to PTX returns to control levels but is still not significantly higher than in P4-alone larvae (SV vs. P4 + PTX, *p* = 0.8906; P4 vs. P4 + PTX, *p* = 0.2013) (Fig. 17C). However, after 30 min, motility in the P4 and PTX cotreatment group is significantly higher than the P4-alone group (P4 vs. P4 + PTX, *p* < 0.0001) (Fig. 17D). Conversely, PTX-treated larvae pre-treated with ORG or P4 still exhibit significantly decreased motility compared to the PTX-alone group (PTX vs. PTX + ORG, *p* < 0.0001; PTX vs. PTX + P4, *p* = 0.0012) (Fig. 17D). Locomotor activity in the PTX + ORG group remained significantly decreased compared to the PTX-alone group and was not significantly different from the ORG-alone group across all time intervals (Fig. 17).

### Inhibition of 5α-reductase using finasteride and effects on motility

**Fig. 18:**
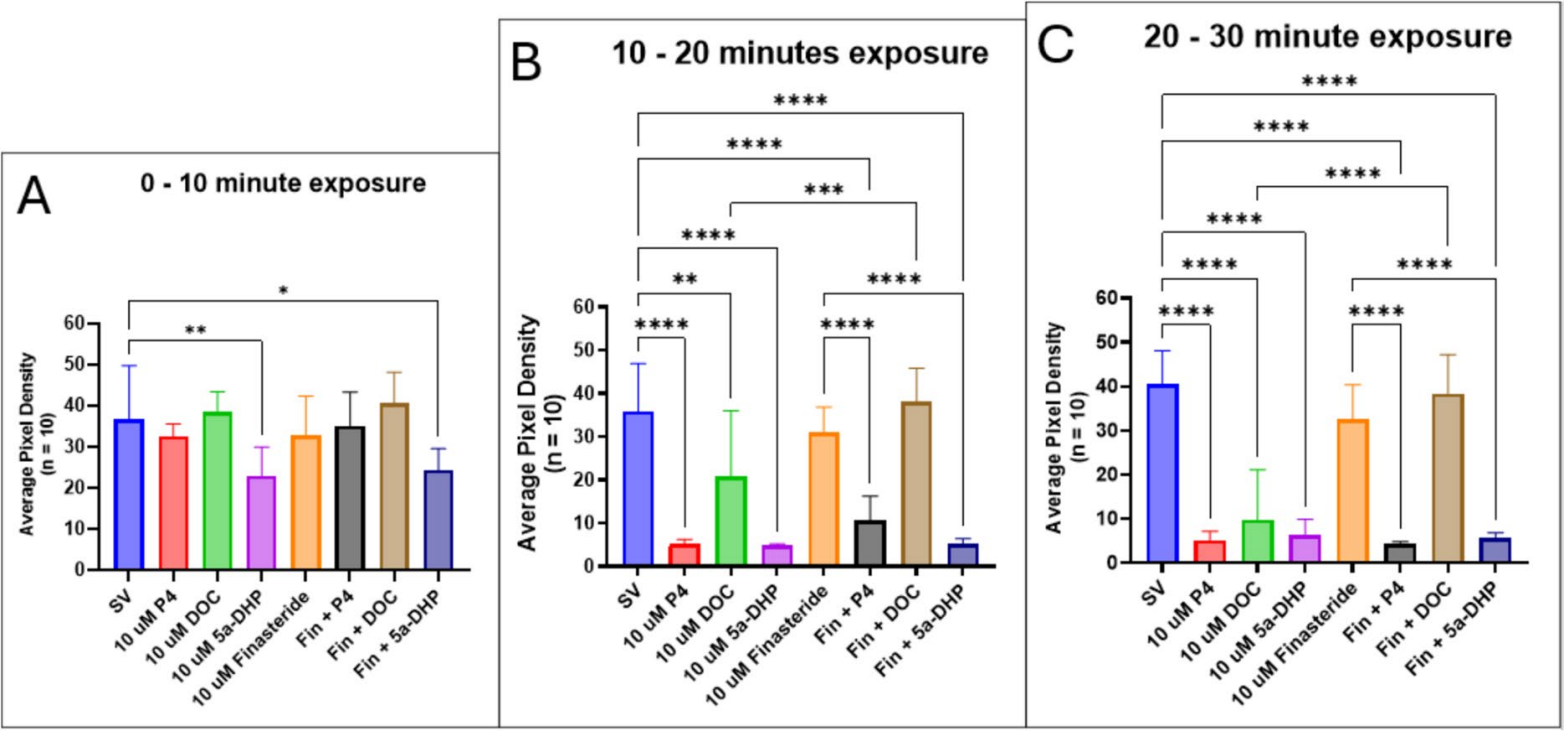
Motility (represented by average pixel density) of 5-dpf zebrafish larvae pre-treated with finasteride for 30 min, then exposed to neurosteroids. Time intervals refer to time after neurosteroid exposure, not finasteride exposure: **A** between 0 and 10 min; **B** between 10 and 20 min; **C** between 20 and 30 min. Values are mean ± SD and were analyzed by one-way ANOVA with Tukey’s post hoc multiple-comparison test. *p < 0.05; **p < 0.01; ***p < 0.001; ****p < 0.0001

The basis of this experiment is that P4 requires the steroidogenic enzyme 5α-reductase to be converted into 5α-DHP, which is further converted into allopregnanolone, a potent PAM of the GABA_A_ receptor, through 3α-hydroxysteroid dehydrogenase (3α-HSD). Finasteride is an inhibitor of 5α-reductase, so this experiment aims to assess whether pre-treatment with finasteride prevents the synthesis of allopregnanolone from P4 and therefore evaluates whether the inhibitory effects of P4 are primarily induced by allopregnanolone and modulation of the GABA_A_ receptor. Similarly, DOC requires 5α-reductase to be converted into DHDOC, which is then reduced to THDOC through 3α-HSD. Locomotor activity of larvae exposed to 10 µM 5α-DHP decreased more rapidly than those exposed to P4 or DOC, with significant reduction of motility occurring within 10 min of exposure to steroids (SV vs. 5α-DHP, *p* = 0.0057; SV vs. P4, *p* = 0.9427; SV vs. DOC, *p* = 0.9996) (Fig. 18A). This decrease also occurred in larvae pre-treated with finasteride and subsequently exposed to 5α-DHP (SV vs. 5α-DHP, *p* = 0.0205) (Fig. 18A). However, after 10 min, locomotor activity is significantly diminished in the P4, DOC, and 5α-DHP-alone groups (SV vs. P4, *p* < 0.0001; SV vs. DOC, *p* = 0.0012; SV vs. 5α-DHP, *p* < 0.0001) and finasteride co-treatment groups with P4 and ORG (SV vs. Fin + P4, *p* < 0.0001; SV vs. Fin + ORG, *p* < 0.0001) compared to the steroid vehicle control group (Fig. 18B). However, the larvae that were exposed to DOC after a finasteride pre-incubation period exhibited similar motility levels as both the steroid vehicle control and finasteride-alone treatments (Fig. 18B). Finasteride alone does not change locomotor activity after 60 min of exposure (equivalent to 30 min after neurosteroid exposure) (SV vs. 10 µM finasteride, *p* = 0.1345) (Fig. 18C). By 30 min post-steroid exposure, both the P4-alone and finasteride + P4-treated larvae had significantly decreased motility compared to the control (SV vs. P4, *p* < 0.0001; SV vs. finasteride + P4, *p* < 0.0001) (Fig. 18C). Additionally, there was no difference in motility between P4-alone and P4-exposed larvae pre-treated with finasteride (P4 vs. finasteride + P4, *p* > 0.9999) (Fig. 18C). Similarly, there was no difference in motility between 5α-DHP-exposed larvae that were and were not pre-treated with finasteride (5α-DHP vs. finasteride + 5α-DHP, *p* > 0.9999) (Fig. 18C). Interestingly, although DOC alone significantly reduces locomotor activity relative to the control (SV vs. DOC, *p* < 0.0001), larvae pre-treated with finasteride before DOC exposure show no such reduction (SV vs. finasteride + DOC, *p* = 0.9957) (Fig. 18C). Larvae pre-treated with finasteride and subsequently exposed to DOC exhibited a recovery in motility to control and finasteride-alone levels.

### Locomotor effects induced by progestogen neurosteroids are not caused by non-specific toxic effects

**Fig. 19:**
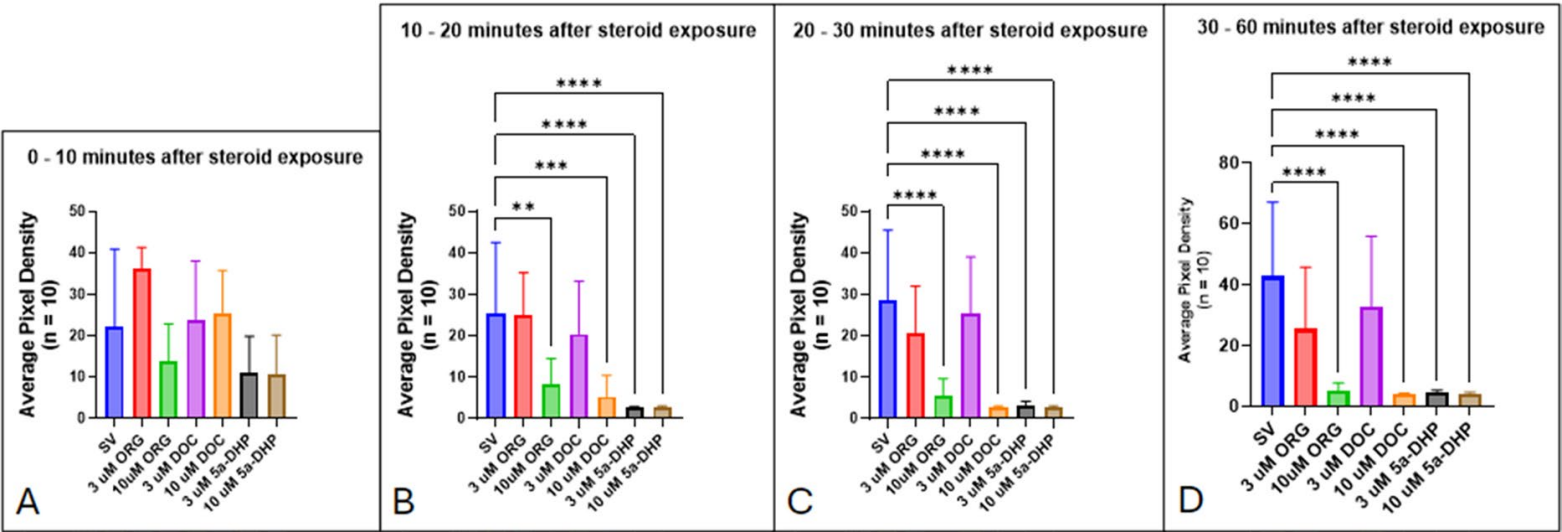
Decreased locomotor activity upon exposure to neurosteroids is shown in stacked image analyses at 10-min intervals: **A** 0 to 10 min, **B** 10 to 20 min, and **C** 20 to 30 min after application. **D** Motility within a 30-min time interval from 30 to 60 min after neurosteroid application. Values are mean ± SD and were analyzed by one-way ANOVA with Tukey’s post hoc multiple-comparison test. **p < 0.01; ***p < 0.001; ****p < 0.0001

To determine whether the decrease in locomotor activity exhibited by zebrafish larvae was caused by an anesthetic-like effect or a toxic effect, a wash experiment was conducted in which 7-dpf zebrafish larvae were exposed to 3 and 10 µM ORG, DOC, and 5α-DHP for 1 h before washing with untreated system water. Significant changes in motility for larvae exposed to neurosteroids were first recorded 10 min after initial exposure (Fig. 19B). Zebrafish larvae showed a stereotypical reduction in locomotor behaviors compared to the control group by 30 min of exposure to 10 µM ORG, 10 µM DOC, and both concentrations of 5α-DHP exposure (Fig. 19C), suggesting a higher sensitivity to the latter. After 30 min, motility was significantly decreased in larvae exposed to 10 µM ORG and DOC and both 3 and 10 µM 5α-DHP (Fig. 19D).

**Fig. 20:**
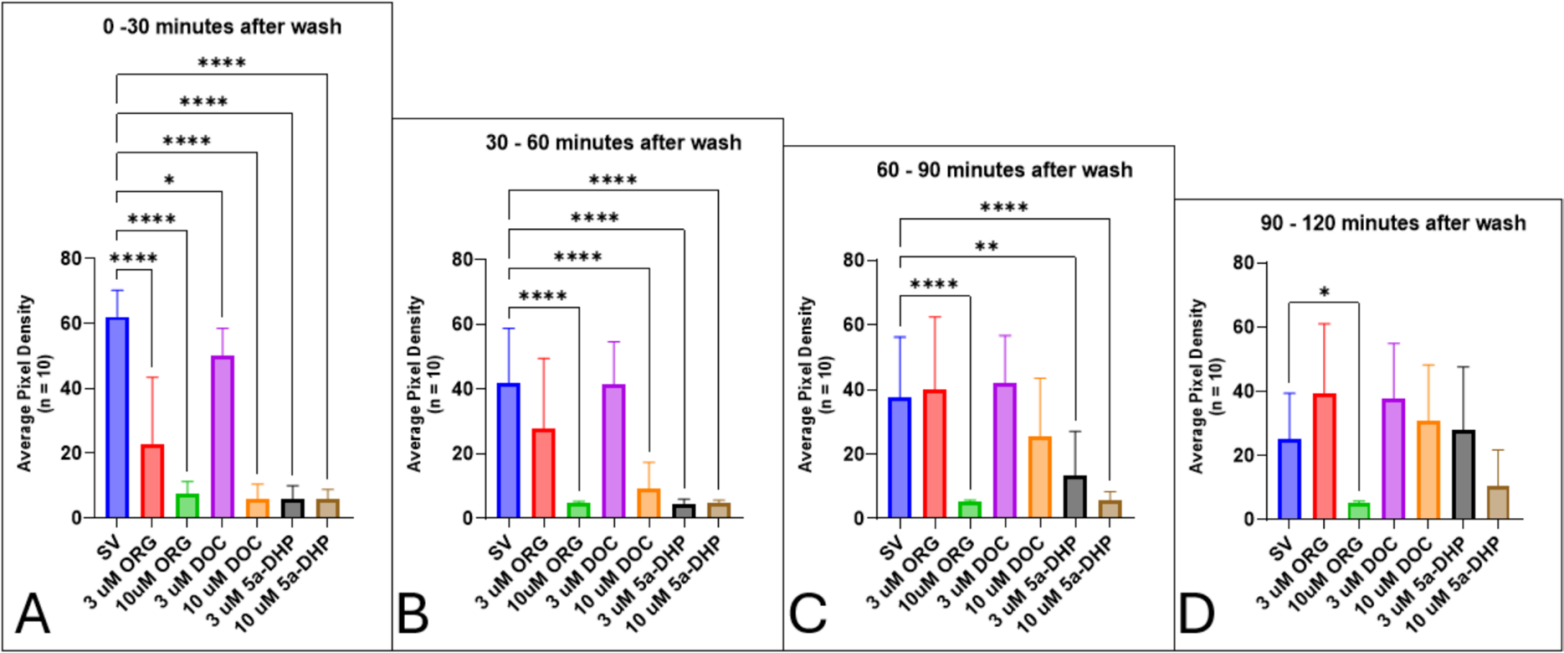
Locomotor activity returns after neurosteroids are washed out and replaced with untreated system water: **A** 0 to 30 min after wash, **B** 30 to 60 min after wash, **C** 60 to 90 min after wash, and **D** 90 and 120 min after wash. Values are mean ± SD and were analyzed by one-way ANOVA with Tukey’s post hoc multiple-comparison test. *p < 0.05; **p < 0.01; ***p < 0.001; ****p < 0.0001

After a 1-h exposure period and measuring the motility of the zebrafish larvae, neurosteroids were removed, and larvae were washed with untreated system water. Imaging of the larvae in untreated system water resumed for up to 26 h after the wash. Within the first 30 min of the wash period, all treatment groups have significantly diminished locomotor behaviors (Fig. 20A). However, after 30 min, larvae in the 3-µM DOC and 3-µM ORG treatment groups show motility that is not dissimilar to the control group (Fig. 20B). Between 60 and 90 min after the wash, both DOC treatment groups and the 3-µM group recover locomotor behaviors to the level of the control group (Fig. 20C). By 120 min after the wash, larvae in all treatment groups except the 10-µM ORG group have recovered locomotor behaviors (Fig. 20D).

**Fig. 21:**
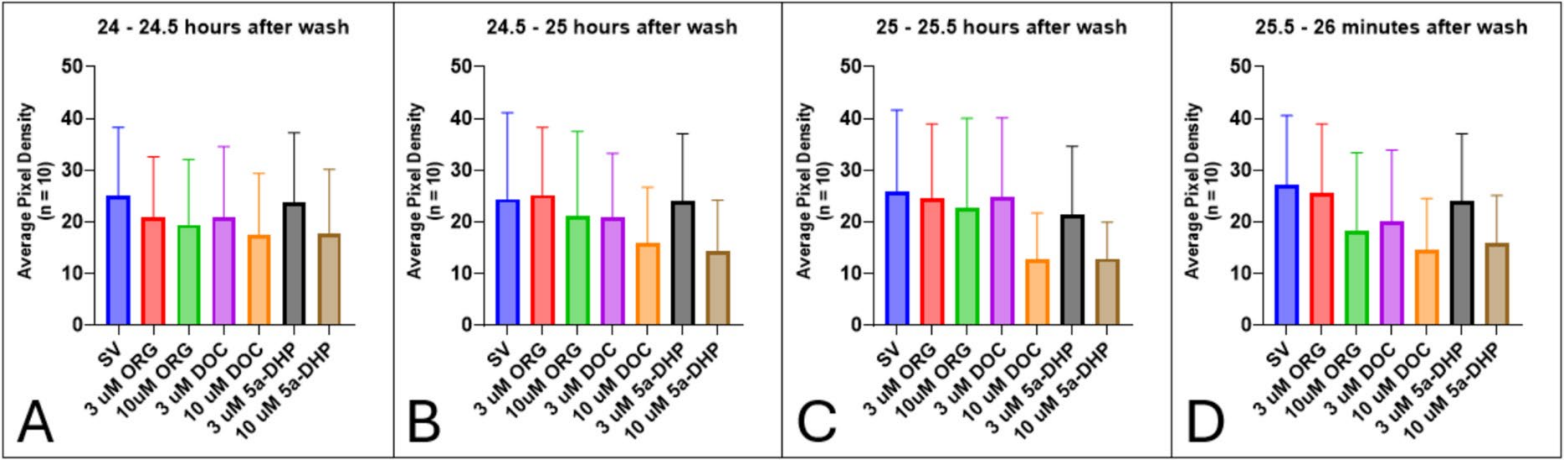
Locomotor activity after a 24-h wash period, shown in 30-min intervals: **A** 24 to 24.5 h after wash, **B** 24.5 to 25 h after wash, **C** 25 to 25.5 h after wash, and **D** 25.5 to 26 h after wash

Imaging ended after 120 min but resumed 24 h after the wash. Figure 21 shows how the locomotor activity of the zebrafish in all treatment groups has recovered when exogenous neurosteroids are removed. After 24 h of being placed back in untreated egg water, motility in all treatment groups is not significantly different compared to the control group (Fig. 21).

### Preliminary studies of mPRα expression across different stages of development and electrophysiological effects of P4 and ORG

Preliminary Western blot and EEG data were obtained that suggest possible mPR and GABA_A_ involvement in facilitating the locomotor effects observed (see supplementary files). At 1 hpf, mPRα was not detected (Fig. S1). Only in zebrafish larvae 3 dpf and older was an immunoreactive band at around 40 kDa present in the Western blot (Fig. S1). The mPRα band intensity increased with the age of the zebrafish, with 5-dpf larvae samples having a darker band than 3-dpf larvae samples. We carried out preliminary evaluations of whether the inhibitory effects of P4 and ORG were based in the brain of 5-dpf zebrafish larvae using an electrode to measure electrical activity through local field potentials from the hindbrain. After an acclimatization period of 5 min after the electrode probe is applied to the head of the larvae, a baseline of electrical activity in the hindbrain, represented by local field potential recordings, was measured to represent the normal brain activity before PTX or P4 is added (Fig. S2). The baseline recording revealed normal brain activity, indicated by an EEG that had few oscillations. This appears as a trace with low frequency and a small number of peaks within a 1-min time period (Fig. S2 A). Larvae were first exposed to 2 mM PTX for 5 min to induce a seizure-like effect in the brain, which causes an apparent increase in brain activity as represented by rapid high-frequency wave oscillations compared to the baseline (Fig. S2B, C). After 25 min of PTX exposure, 30 µM P4 was added (Fig. S2), which caused brain activity to severely drop, and the trace became flatter with no large oscillations compared to the PTX exposure (Fig. S3 C). However, after 1 h of P4 exposure (1 h and 25 min after PTX application), the rapid PTX-like oscillations resumed (Fig. S4D).

The selective mPR agonist ORG was used to determine whether the inhibitory effects on locomotor activity in 7-dpf zebrafish larvae were impacted by diminished brain activity upon exposure to the compound. A similar experimental timeline was used for ORG (Fig. S2). After the 5-min acclimatization period, the baseline recording shows the normal brain activity in the 7-dpf zebrafish before the drugs are added (Fig. S4 A). In this trace, there are very few fluctuations in voltage, as is standard for baseline recordings. After the larvae are exposed to 400 µM PTX for 5 min, an EEG recording shows highly fluctuating local field potentials that have a high frequency, which can suggest an increase in brain activity in the motor region of the brain (Fig. S4). After both 30- and 60-min incubation times with ORG, EEG recordings show an attenuation of local field potentials measured by the electrode probe, where voltage does not fluctuate as it did when PTX was added (Fig. S4 C, D).

## Discussion

Animal behavioral parameters are an informative endpoint to study the neurological effects of pregnane neurosteroids. Therefore, in this study, we investigated the effect of acute exposure to progestogens in zebrafish embryos (24 hpf) and larvae (5–7 days old), specifically on spontaneous tail coiling behavior and swimming activity, respectively. We demonstrated how exposure to exogenous progestogens may produce inhibitory effects on central nervous system activity in larval zebrafish. The data shed light on which receptors may be responsible for progestogen-induced changes in motility. From our results, we cannot rule out the possibility of PNS effects as well. For example, the nicotinic acetylcholine channel was expressed in *Xenopus* oocytes, and progesterone was found to inhibit the action of this receptor (Valera et al. 1992). We were able to quantify motility in larval zebrafish using a recording method that allows for capturing an image of the larvae in 96-well plates at set intervals (either 1 picture every 5 s or 1 picture every 2 min) for a specified duration of time (i.e., several hours), similar to the CAS method described by Lessman (2002). This method provides many advantages, including evaluation of the amount of time it takes a pregnane neurosteroid to inhibit locomotor behaviors and assessing how motility can change over time. Individually, the images captured do not reveal information about neurosteroid-induced motility changes, but when the images are stacked for specified intervals of time and then flattened, the motility patterns can be easily observed. The locomotor behaviors measured in this study were described in terms of APD, which represents motility. For example, lower APD reflects decreased motility due to the anesthetic effects of progestogens. If the fish is sedated and unable to swim around the well, then it will appear only in a single orientation within the well. Therefore, when the images are stacked across a 5-, 10-, or 30-min time interval and the APD is measured, the ROI set around each well will capture the pixel density of the fish remaining in one spot. Thus, the number of pixels within the ROI that represent the larvae will be small. This contrasts with fish displaying higher locomotor activity. Fish that are highly active will move around to different spots in the well sporadically, reflecting a characteristic higher swimming frequency by 5 dpf (Saint-Amant and Drapeau 1998; Drapeau et al. 2002). Therefore, each time an image is captured, the fish will likely be in another spot of the well and oriented differently from the previous position. Since images are captured every few seconds or minutes and then stacked, the fish will appear to cover much of the well. Therefore, the ROI covering the well will detect a higher APD from the body of the active fish covering this area.

Our data show that progesterone does not act through the classical pathway via nPR (Figs. 7 and 8) or GR (Figs. 9 and 10) to decrease locomotor activity but rather may be acting through the GABA_A_ receptor as a PAM or through the mPR to mediate the anesthetic effects observed. This is supported by how quickly the larvae lost locomotor activity after exposure to the steroids (Fig. 2A). This rapid onset of P4-induced anesthesia suggests that these effects are unlikely to be induced through gene transcription but rather mediated by a rapid nongenomic pathway. It also supports the hypothesis that P4 can produce a decrease in locomotor behaviors via activation of the mPR—a target of P4 that acts as a GPCR and utilizes a G-protein to induce an effect (Tang et al. 2005; Thomas et al. 2007; Thomas 2012)—or another membrane-bound receptor such as the GABA_A_ receptor. Additionally, P4-induced decreases in motility do not appear to be caused by a non-specific toxic effect since locomotor activity was recovered upon washing with clean egg water after a 2-h exposure period (Fig. 5). This provides further evidence that progestogens can act as anesthetics through receptors that modulate behavior.

Since nuclear steroid hormone receptors are transcription factors, activation results in binding to hormone response elements, which are specific sequences of DNA, to regulate gene expression and lead to transcription and translation of steroid-dependent proteins (Scarpin et al. 2009; Garoche et al. 2020). These processes make the genomic pathway of nPR activation slower compared to the nongenomic mechanisms employed by surface receptors like the mPR or GABA_A_ receptor. Additionally, despite activation of the nPR using 17α-HP, we recorded no significant effects on locomotor behaviors (Fig. 7). Similar results were found, showing that exposure to 10 µg/L (0.03 µM) 17α-HP does not induce physiological (swimming or muscle contractions) or transcriptional changes in early-stage zebrafish (Fent et al. 2018). However, at higher concentrations (3 and 10 µM), 17α-HP was reported to significantly reduce locomotor activity in zebrafish larvae (Abramova et al. 2023). The contrast with our results may be due to the protocols with which locomotor activity was assessed. Whereas the Abramova study utilized an open field test to measure avoidance behaviors, our experiments measured spontaneous swimming movements and were performed in 96-well plates. Additionally, pre-treatment with RU486 to inhibit the nPR and GR, followed by exposure to P4, resulted in decreased motor activity (Figs. 8 and 10), further suggesting that neither the nPR nor the GR facilitates the anesthetic effects observed when larvae are exposed to P4. Also, previous studies have shown that exposure to P4 through intraperitoneal injection promotes anesthesia in nPR knockout mice, indicating that locomotor activity is not regulated through genomic pathways (Reddy et al. 2004; Reddy and Apanites 2005). Overall, these observations indicate that the anesthetic effects of progestogens are unlikely to be mediated by the nPR or GR but rather by surface receptors that facilitate activity through second messengers or by modulating the activity of receptors rapidly that promote inhibition or excitation.

Interestingly, when zebrafish embryos (24 hpf) were exposed to P4, there was a dose-dependent *increase* in locomotor activity (Fig. 6). Thus, the locomotor effects of P4 in zebrafish embryos contrast starkly with those found in zebrafish larvae. This suggests that the anesthetic effects of P4 are age-dependent. The increase in motility suggests that a poorly understood phenomenon called paradoxical excitation occurs in zebrafish embryos. Paradoxical excitation is a phenomenon in which anesthetics like benzodiazepines produce an excitatory effect on brain and motor activity, resulting in aggressiveness and agitation (Litchfield 1980; Hall and Zisook 1981; Short et al. 1987). There is evidence that paradoxical excitation may be associated with the GABA_A_ receptor (McCarroll et al. 2019), which suggests that P4 and its metabolites may act on the GABA_A_ receptor to modulate locomotor activity. The GABA_A_ receptor utilizes the differential concentrations of extracellular and intracellular Cl^−^ to facilitate ion conductance. In mature neurons, extracellular [Cl^−^] is higher; therefore, GABA_A_ receptor activity induces hyperpolarization by increasing the flow of Cl^−^ into the neuron, causing inhibition of the neuron by decreasing the chance of releasing an action potential. In immature neurons, [Cl^−^] is higher inside the cell than outside; therefore, the GABA_A_ receptor would facilitate efflux of Cl^−^ and increase the chance of depolarization (Blaesse et al. 2009). This can be due to the expression of K^+^-Cl^−^ cotransporters (KCC2) on mature neurons, which function to maintain low cytosolic concentrations of Cl^−^ (Fukuda et al. 1998; Payne et al. 2003; Reynolds et al. 2008). However, immature neurons express higher amounts of the Na^+^-K^+^−2 Cl^−^ 1 co-transporter (NKCC1), which functions in Cl^−^ uptake, and low levels of KCC2, resulting in increased baseline intracellular Cl^−^ levels (Kaila 1994; Fukuda et al. 1998; Alvarez-Leefmans et al. 2001; Ben-Ari 2002; Ben-Ari et al. 2007; Reynolds et al. 2008). Zebrafish embryos undergo primary motor neuron (PMN) axogenesis around 17 hpf, which ultimately innervates the skeletal trunk muscles to stimulate contractions. These PMNs continue to mature after 24 hpf (Myers et al. 1986; Kimmel et al. 1995; Drapeau et al. 2002). The growth and development of the secondary motor neurons follow around 26 hpf (Myers et al. 1986; Drapeau et al. 2002), indicating that the neurons emerging from the spinal cord are not fully developed at the time of our locomotor assay in 24-hpf zebrafish. The excitatory response we observed in zebrafish embryos may be due to immature neurons containing a higher concentration of intracellular [Cl^−^] and lower amounts of extracellular [Cl^−^] (Cherubini et al. 1991; Watanabe and Fukuda 2015). Due to paradoxical excitation from P4 exposure we observed in zebrafish embryos, we focused on locomotor behaviors upon exposure to progestogens in larval-stage zebrafish.

PGRMC1 may work in conjunction with mPR to facilitate physiological effects such as activating the mitogen-activated protein kinase (MAPK) pathway, phosphorylating GABA_A_ receptor (Thomas et al. 2014), or regulating oocyte maturation (Wu et al. 2020). We used a PGRMC1 antagonist called AG205 to evaluate whether PGRMC1 plays a role in modulating locomotor activity. A concentration of 20 µM was used in accordance with a study by Aizen et al. (2018), which reported that treatment with AG205 blocked 17α,20β-DHP-induced maturation of oocytes through mPRα. However, our results show that inhibition of PGRMC1 activity does not affect P4- or ORG-induced loss of motility (Fig. 11), indicating that anesthesia may be mediated by the mPR independent of PGRMC1. To test how activation of the mPR itself affects locomotor behavior, we used the MIS in zebrafish oocytes, 17α,20β-DHP, which acts on mPRα located on the surface of the oocyte to facilitate maturation and preparation for fertilization (Nagahama 1997; Zhu et al. 2003a, b), although mPRβ has also been found to have potent interactions with 17α,20β-DHP (Hanna et al. 2006). Our results show that exposure of larval zebrafish to 17α,20β-DHP did not alter motility compared to the control (Fig. 12). In contrast to this, we found that ORG, a selective mPRα agonist, significantly decreases locomotor activity in zebrafish larvae (Fig. 13). While this is surprising, it is possible that this is due to a different variation of mPRα being activated by ORG, or that ORG may act on a different mPR subtype entirely in zebrafish, which express multiple subtypes of mPR (mPRα 1/2, mPRβ, mPRγ1/2, mPRδ, mPRε) (Tang et al. 2005; Hanna et al. 2006; Ashley et al. 2009). Cellular activities in the oocyte and early embryo are dependent on maternal factors until the embryo reaches the maternal-to-zygotic transition phase of development around 10 hpf (Kane and Kimmel 1993; Wragg and Müller 2016). Therefore, it is possible that the mPRα responsible for oocyte maturation may be expressed from pre-zygotic maternal DNA transferred from the ovary. We showed that 24-hpf zebrafish embryos do not express mPRα (Fig. S1), suggesting that this maternal mPR subtype may be downregulated after fertilization and embryogenesis before zygotic genes start to be transcribed. Additionally, expression analysis of 3 mPR subtypes (mPRα, mPRβ, and mPRγ) reveals that mPRα is more highly expressed in reproductive tissues like the testis and ovaries, while mPRβ was found to be exclusively localized to the brain and spinal cord, and mPRγ was expressed in the kidney and adrenal glands (Zhu et al. 2003a, b). In addition, since mPRs are structurally similar to GPCRs, they may be associated with either an inhibitory or stimulatory G-protein (Zhu et al. 2003a, b). Progesterone and ORG have been shown to activate an mPR-associated inhibitory G-protein (*G*_i_) that acts to downregulate cAMP (Sleiter et al. 2009; Dressing et al. 2012; Castelnovo et al. 2020). Also, studies using nPR-deficient mammalian breast cancer cells, MDA-MB-231, transfected with zebrafish mPRα and mPRβ and exposed to 17α,20β-DHP show that both receptors mediate their effects through activation of the MAPK pathway, which subsequently decreases cAMP levels (Hanna et al. 2006). Interestingly, exposing MDA-MB-231 cells to pertussis toxin (a *G*_i_ inhibitor) blocked mPRα signaling via a *G*_i_ protein, resulting in decreased cAMP levels, but it did not produce the same effect through mPRβ (Hanna et al. 2006; Thomas et al. 2007). Also, experiments using the stimulatory G-protein (*G*_s_) enhancer, cholera toxin, revealed that cAMP levels do not change after activation of mPRα and mPRβ (Hanna et al. 2006). These results suggest that mPRα is coupled to *G*_i_, while mPRβ may be coupled to a different member of the G-protein superfamily. ORG has also been found to be able to induce maturation in oocytes both in vivo and in vitro (Rezanujjaman et al. 2020), which suggests it acts on similar receptors and pathways as 17α,20β-DHP. ORG-induced activation of mPR in mammalian cells has been shown to increase tonic inhibition and phosphorylation of the GABA_A_ receptor through activity of protein kinase A (PKA) and protein kinase C (PKC), which is similar to the effects that allopregnanolone and THDOC have on the GABA_A_ receptor (Modgil et al. 2017; Parakala et al. 2019). Therefore, it is possible that the decrease in motility by ORG (Fig. 13) is facilitated by mPR-mediated phosphorylation of the GABA_A_ receptor and increases tonic inhibition. This could subsequently diminish signals sent along the spinal cord that reach the primary motor neurons and innervate skeletal muscle cells, thereby inhibiting muscle contraction to prevent locomotor activity. Alternatively, P4 and ORG could act directly on the GABA_A_ receptor as PAMs, much like allopregnanolone. The IC_50_ values of ORG on motility were also larger than those of P4 (Table 2), indicating that although ORG has a higher binding affinity to mPRα than P4 (Kelder et al. 2010), the effects on locomotor behaviors are less potent and can suggest P4 may use multiple pathways to induce an anesthetic effect.

DOC significantly reduced locomotor behaviors in zebrafish larvae (Fig. 1), which is consistent with studies showing DOC acts as a PAM on GABA_A_ receptors in rats (Reddy and Rogawski 2002). This is likely due to the conversion of DOC into THDOC, a known PAM of the GABA_A_ receptors, by 5α-reductase and 3α-HSD (Karavolas and Hodges 1990; Reddy and Rogawski 2002). Additionally, we show that exposure to 5α-DHP (the direct precursor to allopregnanolone) results in significantly reduced locomotor activity in zebrafish larvae (Fig. 15). Conversion of 5α-DHP into allopregnanolone is catalyzed by the activity of 3α-HSD (Diotel et al. 2011). Since we observed a significant motility decrease in zebrafish larvae exposed to DOC, which is likely caused by conversion into THDOC by 3α-DHP, it is likely that the same steroidogenic enzyme converts 5α-DHP into allopregnanolone in zebrafish.

The decrease in motility by DOC was reversed when the larvae were subsequently exposed to the GABA_A_ receptor antagonist, PTX. PTX is a GABA_A_ receptor antagonist and prevents Cl^−^ influx and hyperpolarization, leading to seizure-like stimulation of locomotor behaviors in zebrafish larvae (Yang et al. 2017; Bandara et al. 2020). Therefore, since blocking the GABA_A_ receptor using PTX resulted in recovered motility after P4- and DOC-induced loss of motility (Figs. 16 and 17), these data support that DOC (and therefore THDOC) and P4 may act as PAMs on the GABA_A_ receptor to decrease motility. Since motility in the PTX + DOC group matches PTX-alone levels after 20 min (Fig. 17C), this suggests that DOC targets the GABA_A_ receptor to induce inhibition. However, motility in the PTX + P4 treatment group remained significantly lower compared to the PTX-alone group, suggesting it is possible that P4 may act on other receptors to produce an anesthetic effect. The locomotor activity of larvae in the PTX + P4 group also reached control levels after PTX exposure, suggesting that the inhibitory effects of P4 may negate the PTX-based excitation. Additionally, since ORG selectively targets the mPR, the stimulatory effect from PTX can be mitigated by the mPR-facilitated activation of other signaling pathways to decrease motility. This may be through opening more GABA_A_ receptors, targeting extrasynaptic GABA_A_ receptors to increase tonic inhibition (Parakala et al. 2019), or by modulating the activity of excitatory glutamate receptors (i.e., NMDA) (Wang et al. 2007; Giuliani et al. 2011; Vyklicky et al. 2016). Overall, these results suggest that P4 may produce an anesthetic effect through the combined activation of separate pathways.

Finasteride is an inhibitor of 5α-reductase, which is a steroidogenic enzyme that converts P4 into 5α-DHP (the direct precursor of allopregnanolone) and DOC into DHDOC (the direct precursor to the potent neurosteroid THDOC) (Karavolas and Hodges 1990; Celotti et al. 1992; Normington and Russell 1992; Thigpen and Russell 1992; Reddy and Rogawski 2002; Bosse et al. 2021). Finasteride blocks the anti-convulsant activity of P4 during pentylenetetrazol (PTZ)-induced seizures in mice (Kokate et al. 1999), suggesting that blocking 5α-reductase activity prevents the formation of allopregnanolone. Finasteride has been shown to have the same function as a 5α-reductase inhibitor in teleost fish (Lee et al. 2015; Cano-Nicolau et al. 2016). We showed that pre-treatment with finasteride did not prevent P4-induced loss of motility (Fig. 18), suggesting that although finasteride prevented allopregnanolone synthesis, P4 can mediate inhibitory effects through other pathways, such as via the mPR. The mPR has been shown to signal to downstream secondary messengers and phosphorylate the GABA_A_ receptor (Parakala et al. 2019), which can modulate receptor activity (Kittler and Moss 2003). Indeed, mPR-induced phosphorylation of the GABA_A_ receptor by ORG increased tonic inhibition in mammalian cells (Parakala et al. 2019). Additionally, activation of the mPR by ORG boosts GABA_A_ receptor expression (Parakala et al. 2019). This suggests that P4-induced anesthesia can be due to increased tonic inhibition from excess GABA_A_ receptors. Finasteride also prevents the anti-convulsant property of DOC in PTZ-treated rats by blocking the synthesis of THDOC (Reddy and Rogowski, 2002). In contrast to P4, pre-treatment with finasteride prevented a DOC-induced decrease in motility (Fig. 18). This suggests that DOC alone is unable to modulate locomotor behaviors and requires being converted into DHDOC and THDOC. P4 may utilize multiple pathways to inhibit locomotor behaviors, while DOC is only able to induce anesthesia by being converted into THDOC. While we assume that finasteride pre-treatment blocks 5α-reductase activity to prevent the conversion of P4 into 5α-DHP and DOC into 5α-DHDOC based on previous studies, future studies should quantify and confirm this inhibition of conversion.

Preliminary experiments have shown that the zebrafish that have decreased locomotor activity due to exposure to higher doses of progestogen do not recover unless the progestogen is washed out and the fish are submerged in water containing no progestogen. However, we observed that larvae exposed to 1 µM P4 that lost motility between 0 and 1 h and 1 and 2 h displayed no significant difference in locomotor activity after 2 h of exposure. Similarly, when zebrafish larvae are exposed to 1 µM ORG, they show a decrease in locomotor activity within 30 min of exposure; however, after this duration, locomotor activity is no longer significantly different compared to the control. It is worth exploring this concentration with this duration of exposure to further examine the duration and efficacy of P4- and ORG-mediated anesthetic effects. Additionally, while our experiments were performed using 5- and 7-dpf zebrafish larvae, future studies should maintain consistency with the developmental stage of the zebrafish.

We showed that zebrafish larvae are able to recover lost locomotor behaviors when they are immersed in untreated water (Figs. 19–21). This recovery suggests that the loss of motility observed is due to an anesthetic effect rather than a toxic one. While this study focused on anesthetic effects facilitated by modulation of the GABA_A_ receptor and mPR, other receptors such as the NMDA receptor and nicotinic acetylcholine receptor (nAChR) are also targets of neurosteroids (Bullock et al. 1997) and could provide an alternative or additional mechanism for progesterone-induced anesthesia. Motility assays targeting these receptors should be performed to establish a better understanding of how pregnane neurosteroids facilitate anesthetic effects in zebrafish larvae. Future experiments, including motility assays, should consider exploring other pathways of progestogen-mediated motility effects in zebrafish at various stages of development. For example, the nAChR at the neuromuscular junction facilitates the control of muscle contractions (Maconochie et al. 1995; Unwin 2013). P4 and estradiol were found to block nAChR activity by acting as noncompetitive inhibitors on muscle and ganglionic nAChR subtypes (Valera et al. 1992; Lei and Lukas, 1996); therefore, manipulation of nAChR activity can possibly modulate locomotor activity. This can potentially be accomplished through cotreatment assays using α-bungarotoxin or nicotine to block or potentiate nAChR activity, respectively. Another mechanism that could be explored is through the modulation of NMDA receptors. Neurosteroids can modulate the synaptic transmission from NMDA receptors (Korinek et al. 2011; Hansen et al. 2018). For example, pregnenolone sulfate has dual actions on NMDA receptor activity, as it is able to both inhibit and potentiate currents based on the subunits present in the NMDA receptor (Horak et al. 2004). Thus, it is possible that these receptors may play a role in the modulation of locomotor behaviors in progestogen-exposed zebrafish. Due to these receptors being expressed differentially between the embryonic, larval, and adult stages, it will be important to delineate the expression patterns of these receptors and their influence on locomotor activity at these different stages. Also, it would be interesting to observe how the sex of the post-puberty fish would affect their locomotor response to progestogen exposure.

In sum, we demonstrated that P4 may produce anesthetic responses in zebrafish larvae through both the GABA_A_ receptor and the mPR. While we provide preliminary data on EEG effects and mPR presence in test zebrafish, additional future work is required to determine their relevance to pregnane neurosteroid effects. These results provide further insight into how locomotor behaviors during early life stages can be affected by neuroactive compounds in fish. The data in this study provide valuable information that promotes the benefits of using embryonic and larval stage zebrafish as alternative models for studying steroid hormone-induced anesthesia, epilepsy, and anti-convulsant drug screenings. It would be advisable to further experiment with the manipulation of the receptors that modulate behavior by pharmacological or genetic techniques to document the behavioral effects of progestogens.

## Supplementary Information

Below is the link to the electronic supplementary material. 

ESM1 (DOCX 189 KB)

## Data Availability

No datasets were generated or analysed during the current study.
