# Supplementary material for: Effects of progestogen neurosteroids on locomotor activity in zebrafish embryos and larvae
Source: Fish Physiol Biochem. 2025 May 29;51(3):105. doi: 10.1007/s10695-025-01519-6 (PMC12122642; doi:10.1007/s10695-025-01519-6)
Supplement: Supplementary file 1 — (DOCX 189 KB) [file 10695_2025_1519_MOESM1_ESM.docx]

**Effects of progestogen neurosteroids on locomotor activity in zebrafish embryos and larvae**

Supplemental Figures


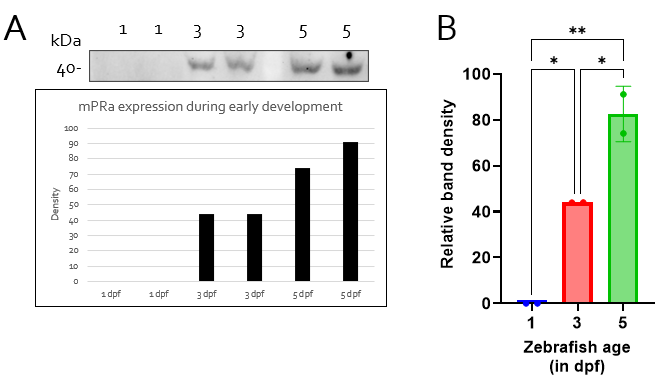


Figure S1. Temporal expression of mPRα in 1, 3, and 5dpf wild-type zebrafish. A) Western blot analysis of whole zebrafish larvae protein extract probed with antibodies against mPRα across developmental stages. Graph shows density of bands relative to the 1 dpf group. B) Quantified expression levels of mPRα in 1, 3, and 53, 5, and 7 dpf groups compared to the 1 dpf group. Data was analyzed using one-way ANOVA followed by post-hoc Dunnett’s multiple comparison test (n = 2), ** p = 0.0098, *** p = 0.0009, **** p < 0.0001.


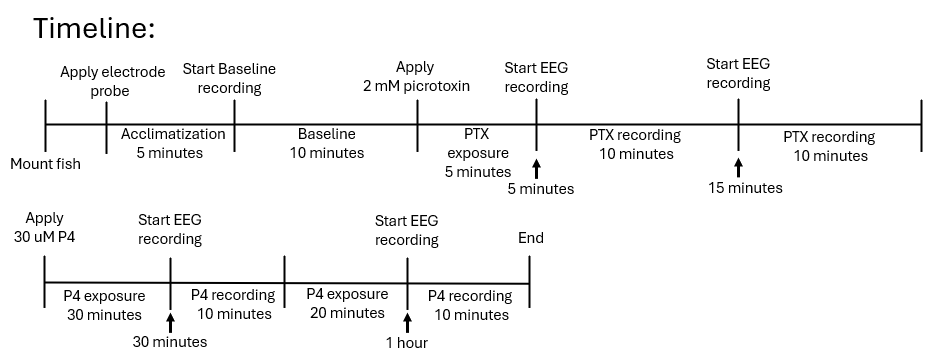
Figure S2. Experimental timeline used for 5 dpf zebrafish larvae exposure to PTX and P4 and EEG recording.


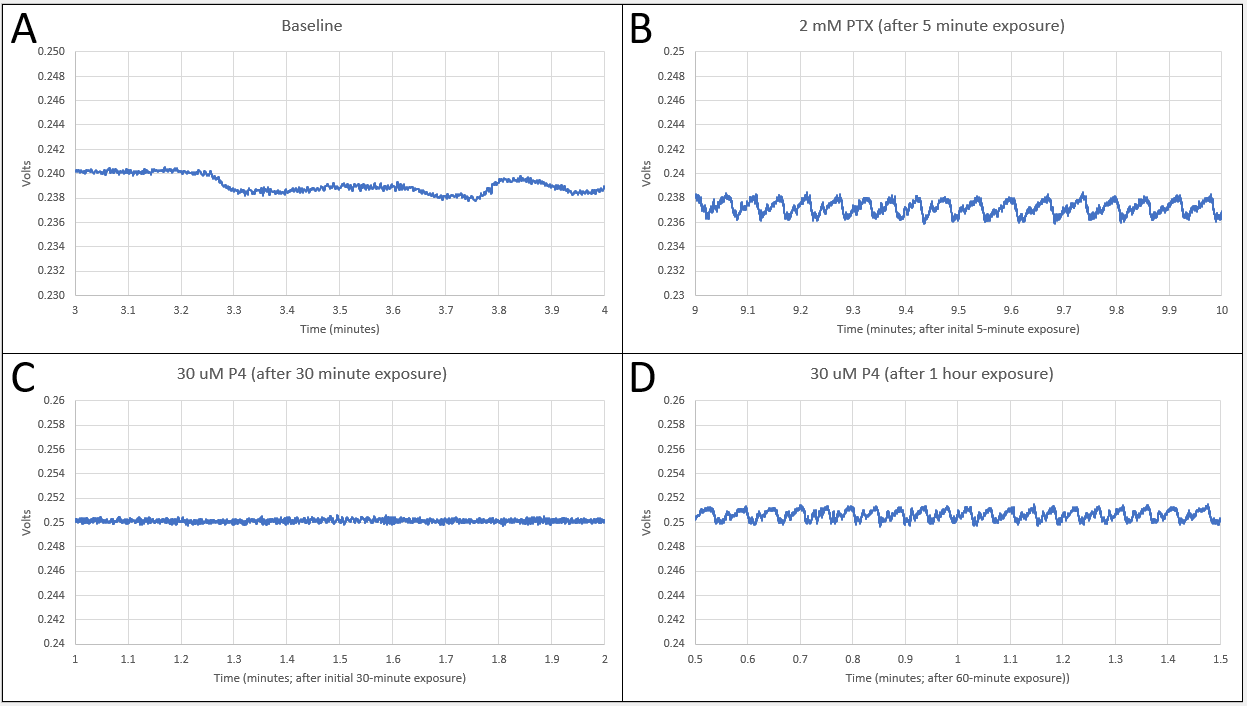


Figure S3. Electrographic activity showing local field potentials over a 1-minute timespan of 5 dpf zebrafish hindbrain upon exposure to PTX then P4. EEG charts have all been scaled to a voltage range of 20 mV and represent 1 minute of total recordings. A) Baseline recording of hindbrain activity before drugs are added. B) Brain activity after a 7-minute exposure to PTX. C) Brain activity 31 minutes after PTX exposure. D) Brain activity is reduced after 60 minutes of P4 exposure.


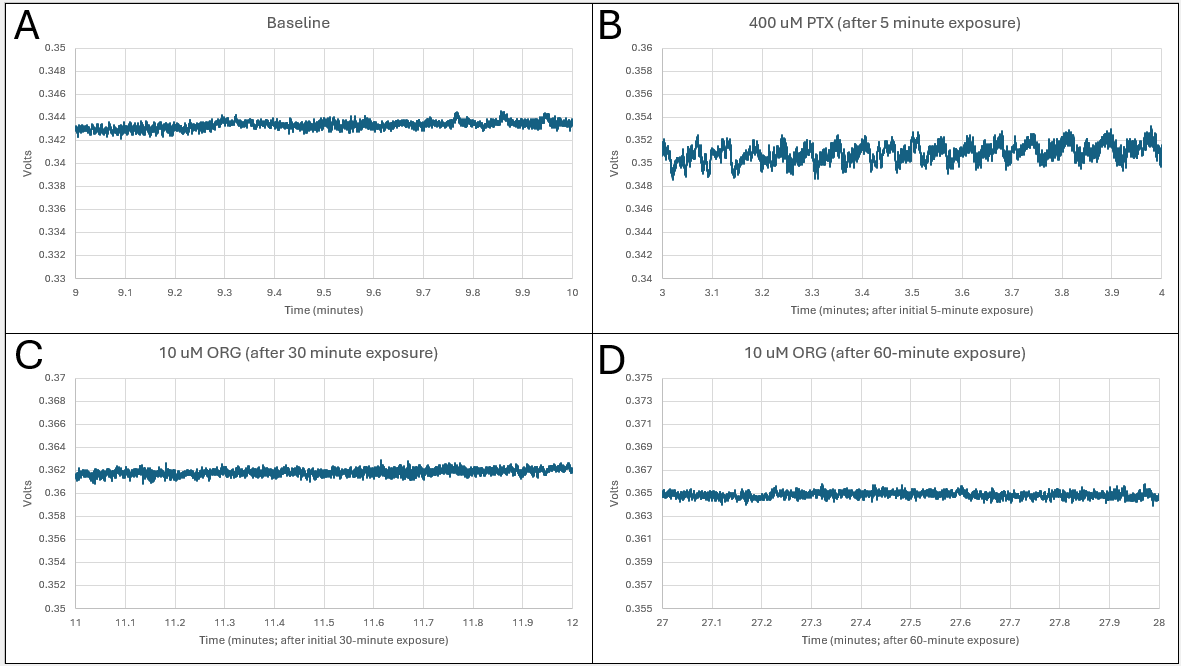


Figure S4. Electroencephalogram recordings of local field potentials from the hindbrain of 7 dpf zebrafish larvae exposed to PTX then ORG. EEG charts have all been scaled to a voltage range of 20 mV and represent 1 minute of total recordings. A) Baseline recording of local field potentials under normal conditions. B) EEG recording 8 minutes after initial PTX application. C) EEG recording of hindbrain activity 41 minutes after initial ORG application. D) EEG recording of hindbrain activity after 87 minutes of ORG exposure.
